# I260Q DNA polymerase β highlights precatalytic conformational rearrangements critical for fidelity

**DOI:** 10.1093/nar/gky825

**Published:** 2018-09-17

**Authors:** Cary Liptak, Mariam M Mahmoud, Brian E Eckenroth, Marcus V Moreno, Kyle East, Khadijeh S Alnajjar, Ji Huang, Jamie B Towle-Weicksel, Sylvie Doublié, J Patrick Loria, Joann B Sweasy

**Affiliations:** 1 Department of Molecular Biophysics and Biochemistry, Yale University, New Haven, CT 06520, USA; 2 Department of Therapeutic Radiology, Yale University School of Medicine, New Haven, CT 06520, USA; 3 Department of Microbiology and Molecular Genetics, University of Vermont, Burlington, VT 05405, USA; 4 Department of Chemistry, Yale University, New Haven, CT 06520, USA; 5 Department of Genetics, Yale University School of Medicine, New Haven, CT 06520, USA

## Abstract

DNA polymerase β (pol β) fills single nucleotide gaps in DNA during base excision repair and non-homologous end-joining. Pol β must select the correct nucleotide from among a pool of four nucleotides with similar structures and properties in order to maintain genomic stability during DNA repair. Here, we use a combination of X-ray crystallography, fluorescence resonance energy transfer and nuclear magnetic resonance to show that pol β‘s ability to access the appropriate conformations both before and upon binding to nucleotide substrates is integral to its fidelity. Importantly, we also demonstrate that the inability of the I260Q mutator variant of pol β to properly navigate this conformational landscape results in error-prone DNA synthesis. Our work reveals that precatalytic conformational rearrangements themselves are an important underlying mechanism of substrate selection by DNA pol β.

## INTRODUCTION

Cellular DNA is under constant insult by various endogenous and exogenous factors, such as reactive oxygen species from cellular respiration and UV radiation from the sun (1). To combat the resultant DNA damage, eukaryotic cells employ a number of different DNA damage repair pathways. The base excision repair (BER) pathway removes 20 000–50 000 lesions per cell per day. Operating mainly upon oxidative and alkylation lesions, BER primarily takes place in the short-patch form, removing single lesions and replacing them with their undamaged counterparts (2,3) (for a review, see (4)). Removal of lesions results in the presence of single-nucleotide gapped DNA (sgDNA). This sgDNA is a substrate for DNA polymerase β (pol β), which catalyzes removal of the deoxyribose phosphate and filling of the single nucleotide gap. For gap filling, pol β is tasked with choosing the correct deoxynucleoside triphosphate (dNTP) from the cellular pool to form a Watson–Crick pair with the templating base. Pol β binds to and covalently inserts the correct dNTP, and the resulting nicked DNA molecule is ligated by a DNA ligase (3), most commonly DNA ligase IIIα. Because numerous intermediates in BER are cytotoxic and possibly mutagenic, this process is very tightly coordinated and regulated, and pol β must be able to faithfully and consistently choose the correct nucleotide when repairing a DNA substrate.

Given pol β’s crucial role of maintaining genome integrity, it is important to understand the mechanism of nucleotide selection, particularly in the context of cancer biology, tumorigenesis and the documented role of pol β in a variety of cancers. Mutations to pol β are observed in over 30% of human tumors in gastric (5), esophageal (6), breast (7,8), bladder (9), lung (10), colorectal (11) and prostate cancer (12). Furthermore, some pol β mutants have been observed to confer resistance to chemotherapy (13,14), marking the polymerase as a druggable target for ancillary chemotherapeutics.

**Figure 1.**
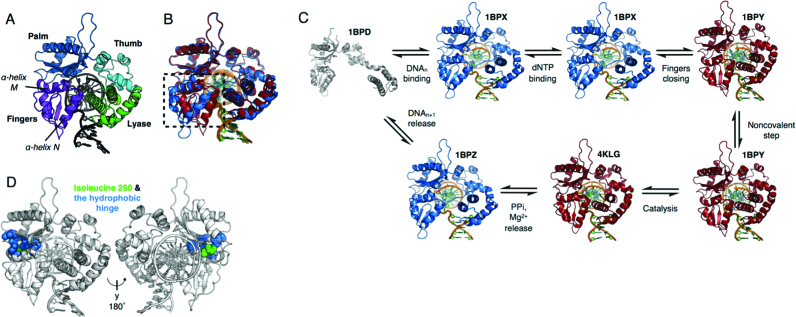
(**A**) Molecular architecture of DNA polymerase β (PDB ID: 1BPY, ternary matched). The lyase domain is shown in green, the thumb subdomain in cyan, the palm subdomain is in blue and the fingers subdomain in purple. (**B**) Overlay of binary open pol β (blue, 1BPX) and matched (Watson–Crick pair) ternary pol β (red, 1BPY) showing the closed conformation. Alpha helix N in the fingers subdomain (marked by the black dashed box) rearranges to form the closed ternary enzyme. (**C**) The hydrophobic hinge shown as spheres, in blue, and isoleucine 260 in green. (**D**) Reaction scheme of pol β in short-patch BER, with PDB codes given for each structure. Crystal structures shown are 1BPD, 1BPX, 1BPY, 1BPZ (17) and 4KLG (23).

Pol β provides an intriguing case study of enzymatic substrate discrimination, one that requires significantly different catalytic properties between very similar ligands based on the identity of the unpaired templating base. In contrast to processive, replicative DNA polymerases, pol β is distributive, generally incorporating single or short segments of nucleotides and has error rates of one in every 3000–10 000 nucleotides incorporated (15). An X family polymerase, pol β, is the smallest eukaryotic DNA polymerase, at 39 kDa and 335 residues. It consists of two primary domains (Figure 1A): the lyase domain and the nucleotidyltransferase domain. The nucleotidyltransferase domain can be further divided into three subdomains analogous to a right hand: the thumb, palm and fingers subdomains (16). sgDNA binding is coordinated by two helix-turn-helix motifs in the thumb and lyase subdomains. The palm subdomain contains catalytic aspartate residues 190, 192 and 256 that are critical for the coordination of magnesium ions, dNTP and the 3′OH of the DNA substrate to enable catalysis (15), while dNTP binding and discrimination are achieved by the fingers subdomain.

Pol β follows an established catalytic pathway, summarized in Figure 1. First, the extended apoenzyme binds sgDNA substrate, causing the apoenzyme to compact around the DNA. In this binary complex, pol β then binds a Mg^2+^ ion and a matched Mg^2+^-dNTP, triggering closure of the fingers subdomain. When this subdomain closes, α-helix N rotates around α-helix M by ∼30° and moves inward by nearly 10 Å to surround the nucleotide in the active site (Figure 1B) (17). While crystallography data (17–20) have structurally defined the various catalytic states (Figure 1C), the mechanism of nucleotide selection remains unclear (21,22). In crystallographic studies, it has been exceedingly difficult to capture a catalytically relevant mispaired state, necessitating that work be conducted using fidelity-reducing divalent cations or other agents that increase dNTP-binding affinity and stabilize the ternary complex (23). For this reason, the true nature of the mispaired complex of pol β with the physiological Mg^2+^ ions bound has remained elusive, and studies conducted on these mispaired states have thus far yielded conflicting results. Some data suggest that the fingers subdomain partially closes with an incorrect dNTP (24), while others suggest that it fails to stably close with an incorrect dNTP (25), resulting in ambiguity as to the role of enzyme domain closure in nucleotide discrimination. Furthermore, some data have suggested that pol β may populate differing conformations when bound to different incorrect dNTPs (22).

Previous studies of DNA polymerase fidelity have suggested that a variety of steps along the polymerase reaction pathway, referred to as ‘kinetic checkpoints’, serve to test the incoming dNTP for complementarity and to reject incorrect dNTPs (26–29). These steps are suggested to involve conformational changes in the enzyme that take place before and after chemistry. One of these conformational changes involves the large fingers domain movement that was discovered when comparing binary (pol–DNA) and ternary (pol–DNA–dNTP) complex crystal structures for several polymerases (Figure 1C). The role of this and other conformational movements of polymerases in substrate specificity is still a matter of debate (20,30–34). Early studies based on thio-elemental effects suggest that this conformational change step precedes chemistry and may be rate-limiting (35,36). Further studies of the kinetic pathways of the Klenow fragment and Klentaq1 (26,37,38), the Y-family DNA polymerase Dpo4 (39) as well as pol β (25) reveal a second non-covalent step that results in further domain rearrangements after fingers subdomain closure, but just prior to chemistry. Kinetic studies with pol β and Taq DNA polymerase I using Forster resonance energy transfer (FRET) and the fluorescence signal from 2-aminopurine suggest that neither the open-to-closed conformational change nor the second non-covalent step is rate-limiting (40–42). Studies with T7 DNA polymerase using a conformationally sensitive fluorophore reveal that the forward rate of the first conformational change is faster than the chemistry step (43). Interestingly, in this study, it is observed that the reverse rate of the conformational change is much slower than the chemistry step, and therefore, only the forward rate of the conformational change and the ground state nucleotide binding constant (*K*_d_) are suggested to be responsible for the specificity for correct nucleotide incorporation.

A potential method to further investigate pol β’s nucleotide selection mechanism is to perturb its ability to discriminate between correct and incorrect dNTPs. A multitude of work has been conducted to identify fidelity compromised and cancer-related mutants of pol β. Interestingly, some error-prone variants of pol β identified in genetic screens (44,45) and a cancer-linked mutation (46) lie within the hydrophobic hinge region of the enzyme. The hydrophobic hinge of pol β is positioned between the palm and fingers subdomains (Figure 1C) and facilitates rotation of the fingers subdomain to close around a matched dNTP. Kinetic studies demonstrate that disruption of the hydrophobic hinge can lead to impaired fidelity, indicating a role for the hydrophobic hinge in ensuring correct nucleotide incorporation (47–51).

Ile260 is located in the hinge region and alteration to Gln (I260Q) results in a mutator pol β enzyme (Figure 1D). Pol β I260Q is a highly active mutant, with catalytic rates (*k*_pol_) nearly identical to those of the wild-type (WT) enzyme (47). However, I260Q exhibits a 23-fold lower level of discrimination during ground state dNTP binding (47). Compared to WT pol β, I260Q exhibits similar affinity for the correct dNTP, but increased affinity for the incorrect dNTP, with a 5-fold lower *K*_d_ value with template A:dATP mismatches than WT (47). The altered substrate-binding affinity of I260Q provides an invaluable opportunity to examine the properties that control substrate-binding selectivity.

To characterize the precatalytic steps of the reaction pathway in an effort to further delineate the underlying mechanism of the reduced fidelity of I260Q, we have employed a combination of FRET analysis, nuclear magnetic resonance (NMR) and X-ray crystallography experimentation. Our work reveals that the I260Q binary complex adopts a partially closed conformation, which resembles the ternary mispaired complex both globally and at the level of individual residues. In combination, our results suggest that precatalytic conformational changes originating from the open binary complex play an important role in substrate discrimination by pol β.

## MATERIALS AND METHODS

### Protein expression and fluorescent labeling of pol β and DNA

**Figure 2.**
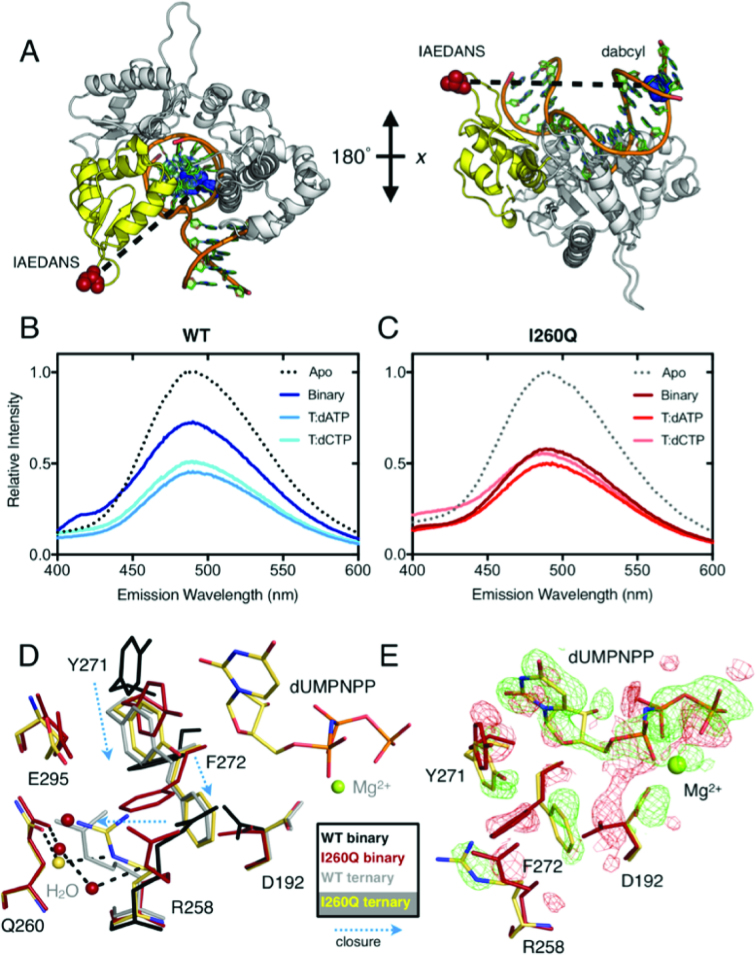
Binary I260Q appears in a semi-closed conformation. (**A**) Location of IAEDANS–dabcyl labels in the dideoxy-terminated V303C pol β construct. Steady-state fluorescence traces for WT (**B**) and I260Q (**C**) binary, matched and mismatched complexes. (**D**) Overlay of I260Q ternary (yellow) and binary (red) with WT ternary (light gray, PDB ID: 2FMS*^19^*) and binary (black, PDB ID: 3ISB*^56^*). Novel water molecules are coordinated by Q260 in both the binary and ternary structures. (**E**) Alignment of I260Q ternary (yellow) and I260Q binary (red) structures, with the isomorphous difference (*F*_o, ternary_ − *F*_o, binary_) map between the two datasets revealing the movements of residues D192, R258, Y271 and F272 upon nucleotide binding in the transition from binary to ternary states. The green map indicates density for the ternary structure and red map indicates density for the binary.

Purification and labeling of I260Q and WT pol β was conducted as previously described (25). The protein was labeled with IAEDANS at position 303 and the DNA with dabcyl, at a position 8 residues upstream of the single nucleotide gap (Figure 2A).

### Generation of DNA oligonucleotides

Deoxyoligonucleotides for the one-base gapped DNA were obtained from the Keck Oligo Synthesis Resource (Yale University) and purified using polyacrylamide gel electrophoresis (PAGE). The desired DNA substrates (Table 1) were annealed in buffer containing 500 mM Tris–HCl (pH 8.0) and 2.5 M NaCl to generate single-base pair gapped DNA as previously described (52,53), but with the ratio of primer:template:downstream oligonucleotides changed to 1:1.2:1.5.

**Table 1.** DNA substrates used in this study

**Name** (length)	Sequence
**Extendable**	5′ GCCTCGCAGCCGGCAGATGCGC_OH_GTCGGTCGATCCAATGCCGTCC 3′
extG (45)	3′ CGGAGCGTCGGCCG**X**CTACGCG**G**CAGCCAGCTAGGTTACGGCAGG 5′
**Non-extendable**	5′ GCCTCGCAGCCGGCAGATGCGC_H_GTCGGTCGATCCAATGCCGTCC 3′
ddG (45)	3′ CGGAGCGTCGGCCG**X**CTACGCG**G**CAGCCAGCTAGGTTACGGCAGG 5′
**Extendable**	5′ GCCTCGCAGCCGGCAGATGCGC_OH_GTCGGTCGATCCAATGCCGTCC 3′
extT (45)	3′ CGGAGCGTCGGCCG**X**CTACGCG**T**CAGCCAGCTAGGTTACGGCAGG 5′
**Non-extendable**	5′ GCCTCGCAGCCGGCAGATGCGC_H_GTCGGTCGATCCAATGCCGTCC 3′
ddT (45)	3′ CGGAGCGTCGGCCG**X**CTACGCG**T**CAGCCAGCTAGGTTACGGCAGG 5′
**NMR substrate**	5′ GGCTGATGCGC_OH_GTCGGTCG 3′
Template G (20)	3′ CCGACTACGCG**T**CAGCCAGC 5′
**NMR substrate**	5′ GGCTGATGCGC_OH_GTCGGTCG 3′
Template T (20)	3′ CCGACTACGCG**T**CAGCCAGC 5′
**X-ray crystallography**	5′ GCTGATGCGC_OH_GTCGG 3′
Template A (16)	3′ CGACTACGCG**A**CAGCC 5′

Bolded bases are the templating bases.

X represents the dabcyl residue.

### Rapid chemical quench experiments

Chemical quench experiments were carried out on a RQF-3 KinTek Chemical Quench Flow apparatus. For WT pol β, a solution of 100 nM protein and 300 nM ^32^P-labeled, one-based gapped extendable DNA (Table 1) were mixed with 100 μM dCTP and 10 mM MgCl_2_. For I260Q, the dNTP was increased to 1500 μM and studied. All mixtures were in 50 mM Tris–HCl (pH 8.0), 100 mM NaCl and 10% glycerol (buffer C) and were allowed to react for 0.02 to 3 s at 37°C. Reactions were quenched with 0.5 M ethylenediaminetetraacetic acid (EDTA) and 90% formamide sequencing dye. Radioactive products were separated on a 20% polyacrylamide gel containing 6 M urea, observed on a Storm 860 Phosphorimager, and quantified based on *n* (substrate) and *n*+1 (substrate with one nucleotide added) products using ImageQuant software. The data were fitted to the following biphasic burst equation using Prism 6 GraphPad software, where *k*_obs_ is the observed rate *k*, while *k*_ss_ is the steady-state rate.

(1) \begin{eqnarray*}\left[ {{\rm product}} \right] = {\left[ E \right]_{{\rm app}}}\ \left( {\frac{{{k_{{\rm obs}}}}}{{{{\left( {{k_{{\rm obs}}} + {k_{{\rm ss}}}} \right)}^2}}}(1 - {e^{ - \left( {{k_{{\rm obs}}} + {k_{{\rm ss}}}} \right)t}} + \frac{{\left( {{k_{{\rm obs}}}{k_{{\rm ss}}}} \right)}}{{\left( {{k_{{\rm obs}}} + {k_{{\rm ss}}}} \right)}}t} \right)\end{eqnarray*}

### Single turnover kinetics

Pol β-AEDANS and radiolabeled extG DNA were assayed on the KinTek rapid quench-flow apparatus to determine *k*_pol_, the maximum rate of polymerization, and *K*_d(dNTP)_ at 37°C in buffer C. Correct nucleotide (dCTP) was titrated from 0.5 to 1500 μM over a range of 0.02 to 10 s with a ratio of pol β: radiolabeled extendable DNA that was empirically determined for single turnover conditions (48). Reactions were quenched with 0.5 M EDTA and 90% formamide dye after incubation for the given time duration. Radioactive products were separated on a 20% polyacrylamide gel containing 6 M urea and quantified as described above. Using Prism 6, the data from each dNTP concentration were fitted to the single exponential equation

(2) \begin{equation*}\left[ {{\rm product}} \right] = \ A\left( {1 - {e^{ - {k_{{\rm obs}}}t}}} \right).\end{equation*}

The *k*_obs_ from these fits were plotted versus [dNTP] and fitted to the hyperbolic equation

(3) \begin{equation*}{k_{{\rm obs}}} = \frac{{{k_{{\rm pol}}}\left[ {{\rm dNTP}} \right]}}{{{K_{{\rm d}({\rm{dNTP)}}}} + \left[ {{\rm dNTP}} \right]}},\ \end{equation*}

where *k*_obs_ is the observed rate constant at each concentration of dNTP, *k*_pol_ is the maximum rate of polymerization and *K*_d(dNTP)_ is the apparent equilibrium dissociation constant for the incoming dNTP. For incorrect dNTP single turnovers (dATP), reactions that took >40 s were quenched manually and were titrated over a concentration range of 10–4000 μM. Reactions were performed in duplicate and are reported as mean values ± the standard deviation.

### Steady-state fluorescence

The fluorescence of pol β WT- or I260-AEDANS was monitored at room temperature on the Photon Technology International spectrofluorometer in 50 mM Tris–HCl (pH 7) and 10 mM MgCl_2_ (buffer D). The sample was excited at 336 nm and an emission scan was performed from 400 to 650 nm. The non-extendable dideoxy template G dabcyl-DNA substrate was added to the protein mixture and the fluorescence was monitored following the addition of 100 μM correct dCTP or 500 μM incorrect dATP. Dilutions after each addition were accounted for in the final analysis.

To correlate our fluorescence data to the distance between our two fluorescent probes, we calculated the efficiency of energy transfer (*E*_FRET_) between the IAEDANS-labeled V303C and dabcyl-labeled DNA using Equation (4) (54).

(4) \begin{equation*}{E_{{\rm FRET}}} = \ 1 - \frac{{{F_{{\rm DA}}}}}{{{F_{\rm D}}}}\end{equation*}

Here, *F*_DA_ is the emission of IAEDANS at 490 nm in the presence of dabcyl-DNA, and *F*_D_ is the fluorescence of IAEDANS in the absence of dabcyl-DNA. The efficiency was then used in Equation (5) to estimate the distance (*r)* separating the two fluorophores.

(5) \begin{equation*}{E_{{\rm FRET}}} = \frac{{{R^6}}}{{\left( {{R^6} + {r^6}} \right)}}\ \end{equation*}

In Equation (5), *R* is the Förster radius, defined as the distance at which energy transfer is 50%. This distance was estimated to be 37.76 Å using Equation (6):

(6) \begin{equation*}R\ = \ 9.78\ \times {10^3}{\left( {{\kappa ^2} \cdot {\eta ^{ - 4}} \cdot {f_{\rm d}} \cdot J} \right)^{{\raise0.7ex\hbox{$1$} \!\mathord{\left/ {\vphantom {1 6}}\right.} \!\lower0.7ex\hbox{$6$}}}},\end{equation*}

where *κ^2^* is the relative orientation of the transition dipoles of the probes and is assumed to be equal to 2/3 for a dynamic random average, *η* is the refractive index, assumed to be 1.344 in a solution of Tris–HCl (55), *f*_d_ is the fluorescence quantum yield of IAEDANS in the absence of dabcyl, assumed to be 0.7 (56) and *J* is the spectral overlap integral in units M^−1^cm^3^, which was measured and calculated using Equation (7):

(7) \begin{equation*}J\ = \ \smallint {E_{\rm D}}\left( \lambda \right){\varepsilon _{\rm A}}\left( \lambda \right){\lambda ^4}d\lambda. \end{equation*}

We measured *E*_D_, the maximum normalized emission of IAEDANS and *ϵ*_A_, the extinction coefficient of dabcyl at each wavelength (*λ*).

### Stopped-flow Förster resonance energy transfer

All experiments were conducted on an SX-20 Stopped-Flow Spectrometer (Applied Photophysics) with samples excited at 336 nm and emission filtered with a 400 nm filter. The temperature was set to 37°C and the voltage was set between 400 and 500 V such that the emission recorded with buffer was roughly 1.5–2.0 V and was kept consistent throughout measurements. Data were collected using the pre-trigger setting for 10 s. The instrument dead time is 2 ms and initial mixing artifacts were calculated based on a test reaction as previously described (57) and were subtracted from the data prior to global fitting. Reaction experiments were set up with 500 nM pol β, 200 nM extendable or dideoxy terminated DNA, and 10 mM MgCl_2_ in buffer C and mixed with an equal volume of solutions containing various concentrations of dNTP and 10 mM MgCl_2_ in buffer C.

To measure the rates of the reverse conformational changes, a dideoxy terminated DNA primer (ddDNA) was used. A pre-formed ternary enzyme–ddDNA–dNTP complex (500 nM pol β, 200 nM ddDNA and 1 μM correct dCTP) was rapidly mixed with 10-fold (5 μM) excess of an unlabeled enzyme-extendable DNA complex.

### KinTek Explorer modeling

Fluorescence traces were analyzed using the KinTek Global Explorer modeling software in order to obtain rate constants as described previously (58,59). Parameters used to constrain our model include chemical quench data as well as previously published stopped-flow rates (25). We constrained the rate constant *k*_1_ ratio (*k*_-1_/*k*_+1_) = *K*_d(DNA)_, which represents the DNA binding event that occurs prior to the beginning of our stopped-flow measurements and set up the simulation to pre-incubate E + D as a first mixing step. *K*_d(DNA)_ was determined using electrophoretic mobility shift assays that yielded values between 1.0 and 2.3 nM for WT and 4.5 and 7 nM for I260Q with template G DNA. We also constrained the rate constant *k*_2_ (*K*_d(dNTP)_), which represents the dNTP binding event from chemical quench data that yielded a *K*_d(dNTP)_ of 1.5 μM for WT and 0.6 μM for I260Q for G:dCTP correct complexes. Additionally, we constrained *k*_+5_, which represents phosphodiester bond formation and corresponds to the rate of chemistry, *k*_pol_, that we measure in rapid chemical quench flow assays (Table 2). The reverse *k*_-5_ was also set to 0 with the assumption that the reverse reaction of chemistry does not take place in our reaction system, in addition to constraining the reverse rates, *k*_-3_ and *k*_-4_, which we measured directly using a trapping experiment.

**Table 2.** Single turnover kinetic data for WT and I260Q pol β on single-nucleotide gapped DNA

Sequence^a^	Protein	*k*_pol_ (s^−1^)	*K*_d(dNTP)_ (μM)	*Dk*_pol_^b^	*DK_d_*_(dNTP)_^c^	Efficiency^d^ (μM^−1^s^−1^)	Fidelity^e^	*X*-fold^f^
G:dC	WT	12.0±0.9	1.5±0.6			8		
	I260Q	3.6±0.08	0.6±0.1			6		
G:dA	WT	0.134±0.008	365±59	90	243	3.7 × 10^−4^	21623	
	I260Q	0.095±0.004	16±3	38	27	5.9 × 10^−3^	1011	21.4
T:dA	WT	12.2±0.6	5.8±0.9			2.1		
	I260Q	5.5±0.21	1.9±0.3			2.9		
T:dC	WT	0.14±0.01	427±66	87	72	3.3 × 10^−4^	6364	
	I260Q	0.09±0.003	11.5±2.5	61	6	7.8 × 10^−3^	372	17.1

^a^The primer-template is extG or extT, with templating base G or T; templating base:incoming dNTP is shown.

^b^Discrimination of *k*_pol_*= k*_pol_(correct)*/ k*_pol_(incorrect).

^c^Discrimination of *K*_d(dNTP)_ = *K*_d(dNTP)_(incorrect)*/ K*_d(dNTP)_(correct).

^d^Efficiency = *k*_pol_/*K*_d(dNTP)._

^e^Fidelity = (correct efficiency + incorrect efficiency)/incorrect efficiency.

^f^ *X*-fold = WT fidelity/I260Q fidelity.

### Crystallization and X-ray data collection

The DNA sequence context used in crystallization is as described in Table 1. The oligonucleotides used in crystallization were synthesized by Midland Certified Reagent Co. (Midland, TX), PAGE purified and mixed in a 1:1:1.2 (primer:template:downstream oligo) ratio. The oligonucleotides were annealed by heating to 90°C for 10 min, allowing to cool to room temperature, and then incubating on ice for 10 min prior to use. The binary complex of I260Q bound to the single-nucleotide gapped DNA substrate was prepared by mixing 250 μM pol β and 300 μM DNA. Crystallization wells contained 18–20% PEG 3350 with 50 mM HEPES pH 7.5, 175–250 mM sodium acetate, 2 mM TCEP and 1% tert-butanol and were incubated at 18°C. Cryoprotection was achieved by bringing the final PEG 3350 concentration to 20% in conjunction with the addition of 14% ethylene glycol. A 30-min soak in cryoprotection reagent for the binary complex was achieved with the addition of 100 mM NaI. Ternary complexes containing the non-hydrolyzable analog dUMPNPP (1 mM) and 50 mM MgCl_2_ were soaked for 1 h. Crystals were flash cooled in liquid nitrogen and data collected on a Bruker D8 Kappa Quest (Bruker-AXS Inc., Madison, WI, USA) diffraction system utilizing a Photon 100 detector. Crystallographic data were processed using Proteum3 (Bruker-AXS Inc., Madison, WI, USA). High-resolution data for the ternary complex were collected at beamline 23ID-B (Advanced Photon Source at Argonne National Laboratory, Lemont, IL) utilizing a Mar300 CCD detector.

### Structure solution and refinement

The I260Q ternary complex with dUMPNPP was solved by molecular replacement using Phaser (60) within the CCP4 (61) suite using a WT ternary model (PDB ID: 2FMS (62)) separated into the four structural domains. Isomorphism comparison was also performed and showed a cross *R* of 43% on intensities (25.8% on amplitudes). A preliminary model was generated with an *R* factor of 37.2 with palm domain (*Z*-score 15.9), lyase domain (*Z*-score 30.9), thumb domain (*Z*-score 39.4) and fingers domain (*Z*-score 42.1). In parallel, data were evaluated for use in isomorphous replacement with the binary iodide soak data. Initial stages of model building were carried out in the absence of the fingers domain, DNA, the hydrophobic hinge region and the incoming nucleotide, utilizing density-modified maps generated using SOLVE (63). The binary iodide soak complex was solved by isomorphous replacement using the I260Q ternary model with a cross *R* on intensities of 39.1% (25.3% on amplitudes) (Supplementary Table S1). Initial models were generated by removing the fingers domain as well as the DNA, incoming nucleotide, waters and metals from the ternary model and performing a rigid body refinement. The binary iodide map showed adequate residual density in the fingers domain. In previous higher resolution structures of pol β, three bound chloride ions have been observed, while in some structures these sites are occupied by water molecules (62,64). Isomorphous and anomalous difference Fourier maps revealed two sites had exchanged for iodide in the binary structure. The ternary dataset showed adequate residual density for the dUMPNPP, and both datasets showed unambiguous density for the hydrophobic hinge region, including the Q260 mutation (Supplementary Figure S1). Models were built using Coot (65) and refined using Phenix (66) with >98% of residues in Ramachandran favored regions as determined by MolProbity (66). Figures were generated using PyMOL (67).

### NMR samples

All NMR work described here was performed with the thermostable C267A mutant of pol β (68) due to WT pol β’s limited stability at NMR concentrations. All samples were ^1^H-^13^C isoleucine, leucine and valine methyl labeled (69) and fully deuterated at all other non-exchangeable positions to increase sensitivity, reduce spectral complexity and simplify data analysis.

Fast-protein liquid chromatography was performed to purify pol β as described previously (70). Samples were equilibrated into NMR buffer (50 mM HEPES, 100 mM KCl, 2 mM dithiothreitol (DTT), 10 mM MgCl_2_ and 10% D_2_O, pH 7.4) using Amicon 10 000 Da molecular weight cutoff spin filters (EMD Millipore, Darmstadt, Germany) or Slide-A-Lyzer dialysis cassettes (Thermo-Fisher, Waltham, MA) to a final concentration of 300–500 μM prior to NMR studies. Nucleoside triphosphate analogs used in this work have a methylene group between the α and β phosphates, inhibiting catalysis by the polymerase (Supplementary Figure S2). All NMR data shown here were collected at 600 MHz (14.1 Tesla), using an HMQC pulse sequence (71). Radio-frequency carrier frequencies were set to 19.5 and 0.75 ppm for carbon and proton, respectively. Additionally, all spectra were internally referenced to the apo I260Q spectrum, at the valine-221 resonance. Valine 221 exhibits minimal chemical shift changes upon DNA or dNTP ligand binding. ILV residues shown here are not stereospecifically assigned, and thus residues with multiple resonances in data analysis appear twice. Isoleucine (ILV) assignments can be seen in Supplementary Figure S3.

NMR data were processed using NMRPipe (72) and Sparky 3 (73), and chemical shift changes described here were considered significant if they were of a magnitude higher than 0.025 ppm, which is 1.5 times the average combined chemical shift changes between 8 WT and 4 I260Q ILV spectra of the apoenzyme. Combined chemical shift changes were calculated using the following equation:

(8) \begin{equation*}\Delta {\delta _{{\rm combined}}} = \sqrt {\frac{{\Delta {\delta _{\rm H}}^2 + \frac{1}{4}\Delta {\delta _{\rm C}}^2}}{2}}. \end{equation*}

In Equation (8), carbon chemical shifts were scaled as per (74).

For the chemical shift vector analyses of the data in this work, the following equations were used for:

(9) \begin{equation*}{\rm{Vector}}\,{\rm{magnitude}} = \sqrt {\Delta {\delta _{\rm H}}^2 + {{\left( {0.2514*\Delta {\delta _{\rm C}}} \right)}^2}} \end{equation*}

(10) \begin{equation*}{\rm{Normalized}}\,{\rm{magnitude}}\,\Delta {\delta _n} = \frac{{\Delta {\delta _{{\rm experimental}}}}}{{\Delta {\delta _{{\rm reference}}}}}\end{equation*}

(11) \begin{equation*}{\rm Cos}\,\,\theta \ = \frac{{{\delta _{{\rm experimental}}}\ \cdot \ {\delta _{{\rm reference}}}}}{{\Delta {\delta _{{\rm experimental}\ }}\times \ \Delta {\delta _{{\rm reference}}}}}\end{equation*}

(12) \begin{eqnarray*}{\rm R}{\rm MS}{{\rm D}_{1.0}} = \sqrt {\frac{{\mathop \sum \nolimits_{i = 1}^n \left( {{{\left( {1 - {\rm cos}\,{\theta _{{\rm meas}}}} \right)}^2} + {{\left( {1 - \Delta {\delta _n}_{{\rm meas}{\rm }}} \right)}^2}} \right)}}{n}} \ \end{eqnarray*}

In Equation (12), *n* is the number of individual chemical shift measurements.

Carr-Purcell-Meiboom-Gill (CPMG) experiments run here were conducted at 600 MHz. Dispersion curves were fit to the following equation:

(13) \begin{equation*}{{{R}}_{\rm{2}}}\left( {\frac{{\rm{1}}}{{{{\rm{\tau }}_{{\rm{cp}}}}}}} \right){{\ = \ R}}_{\rm{2}}^{\rm{0}}{\rm{\ \ + \ }}{{{R}}_{{\rm{ex}}}}\left[1 - \frac{{{\rm{2\,tanh}}\left( {\ \frac{{{{{k}}_{{\rm{ex}}}}{{\rm{\tau }}_{{\rm{cp}}}}\ }}{{\rm{2}}}} \right)}}{{{{{k}}_{{\rm{ex}}}}{{\rm{\tau }}_{{\rm{cp}}}}}}\right],\end{equation*}

where ${{{R}}_{{\rm{ex}}}}{\rm{\ = \ }}{{{p}}_{\rm{a}}}\ {{{p}}_{\rm{b}}}{\rm{\Delta }}{{\rm{\omega }}^{\rm{2}}}{\rm{\ /\ }}{{{k}}_{{\rm{ex}}}}$ , *p*_a_/*p*_b_ are equilibrium populations, *k*_ex_ is the rate of chemical exchange between the two sites and Δ*ω* is the difference in chemical shift between the two sites. In this work, residues were deemed ‘flexible’ if their dispersion curves fit preferentially to Equation (13) over a straight line with a slope of 0, and if they exhibited *R*_ex_ values >2.

DNA substrates for NMR studies were purchased from Integrated DNA Technologies (IDT, Coralville, IA) and annealed in a polymerase chain reaction thermocycler. The annealing protocol is as follows: 95°C for 5 min, 90°C for 3 min, 85°C for 3 min, 80°C for 3 min, 75°C for 3 min, 70°C for 3 min, 65°C for 3 min, 60°C for 3 min, 55°C for 20 min and then held at 4°C until removal from the thermocycler. Proper annealing of DNA substrates was verified using PAGE and the observation of reproducible WT binary NMR spectra. DNA sequences used are seen in Table 1.

## RESULTS

### IAEDANS-labeled WT and I260Q display pre-steady-state burst activity

WT and I260Q were assessed for pre-steady-state burst activity using the extG DNA substrate (Table 1). The fast phase burst rate observed for WT (9 s^−1^) is similar to the burst rate of I260Q (12 s^−1^), indicating that the overall rate of DNA synthesis catalyzed by pol β was not affected by mutation or fluorophore labeling (Supplementary Figure S4). There was, however, a 4-fold difference in the slow phase, which occurred at 1.2 s^−1^ for WT and 0.3 s^−1^ for I260Q.

### I260Q has lower fidelity than WT pol β

Previous work demonstrated that I260Q has lower fidelity than WT pol β, but the enzymes were not labeled with IAEDANS (47). Therefore, we performed fidelity experiments under single turnover conditions with labeled protein and DNA (Supplementary Figure S5). As shown in Table 2, the *k*_pol_ for I260Q (3.6 s^−1^) is ∼3-fold lower than that of WT (12.0 s^−1^). The *K*_d(dNTP)_ of correct dCTP binding opposite template G is similar for both WT (1.5 μM) and I260Q (0.6 μM), indicating that they both bind the correct dNTP with similar affinities. However, the kinetic basis for the increased ability of I260Q to misincorporate nucleotides opposite template G is due predominantly to deficient substrate discrimination at ground state binding. This discrimination, defined as *K_d_*_(dNTP)_(incorrect)*/K_d_*_(dNTP)_(correct), shows that I260Q has >10-fold loss in discrimination in ground state dNTP binding (Table 2). Although the rates of incorporation of incorrect dATP (*k*_pol_) are similar for WT (0.1339 s^−1^) and I260Q (0.095 s^−1^) under these conditions, I260Q binds the incorrect dATP nucleotide more tightly (*K_d_*_(dNTP)_I260Q = 16 μM compared to *K_d_*_(dNTP)_WT = 365 μM) and leads to a higher catalytic efficiency for incorporation of incorrect dNTP substrate by I260Q. Thus, with template G, I260Q has a significantly lower fidelity than WT pol β and is 21 times more likely to insert the incorrect dATP opposite a G compared to WT. The kinetics of pol β with T as the templating base are shown in Table 2. The calculated *k*_pol_ value for incorporation of dATP opposite template T is 2.2-fold lower for I260Q than for WT pol β. However, for incorporation of incorrect dCTP opposite template T, *k*_pol_ values (Table 2) are similar for WT and I260Q. The binding affinity for correct dNTP is ∼3-fold lower for I260Q as opposed to WT pol β, whereas I260Q binds the incorrect dCTP ∼35-fold more tightly than does WT enzyme (Supplementary Figure S6). In addition, I260Q binds the incorrect nucleotide only 6-fold weaker than it does the correct nucleotide compared to a 150-fold difference for WT. The combined impairment of kinetic and binding discrimination leads to fidelity values of 17 times less for I260Q when compared to WT.

### I260Q forms a closed binary structure

To examine the conformational states of WT and I260Q pol β, steady-state FRET experiments were conducted as previously described (25). Pol β was labeled with the fluorescent dye IAEDANS at an engineered cysteine residue at position 303 (V303C), and the DNA substrate was labeled with dabcyl, a quencher for IAEDANS, at a thymine position, 8 residues upstream of the double-strand gap (Table 1, Figure 2A). In this system, FRET signal from the IAEDANS label is increasingly quenched as the distance to the dabcyl molecule decreases. As observed in Figure 2B and C, the fluorescence intensity decreases from apo, to binary, to ternary complexes, in agreement with X-ray structural data (17). In these steady-state FRET experiments, enzyme closure is readily observed in WT correctly paired complexes, as the label moves toward the quencher from a distance of 43.9 Å in the binary to a FRET distance of 36.4 Å in the template T:dATP ternary correct complex. In WT pol β, the ternary complex with correct dNTP exhibits reduced fluorescence compared to the ternary complex with the mispair (36.9 Å), indicating additional closure in the enzyme. This observation is in agreement with prior fluorescence studies (25). Unlike the WT enzyme, binary I260Q has a fluorescence profile similar to the ternary complex with the incorrect dNTP, with a FRET distance of 39.8 Å for the binary compared to 39.1 Å for the I260Q ternary incorrect complex. Similar results are observed for template G (Supplementary Figure S7). FRET efficiencies and the calculated interprobe distances are provided in Supplementary Figure S7.

### The binary complex of I260Q adopts a partially closed fingers conformation in the crystal structure

Our steady-state FRET results suggest that the I260Q enzyme adopts a partially closed conformation when bound to DNA and that it does not completely close in the presence of the correct dNTP. To further characterize the binary I260Q complex, we solved the crystal structure of I260Q in complex with DNA. The dataset collected from a binary complex containing 0.2 M iodide in the cryoprotection reagent (2.25 Å) is the most isomorphous with the ternary I260Q model with dA in the templating position and dUMPNPP as the incoming nucleotide. Model building and refinement of this structure reveals that the I260Q binary complex more closely resembles a typical ternary (RMSD 0.49 Å) rather than a binary complex (RMSD 1.22 Å) (Supplementary Figure S8). Isomorphous difference maps generated between the I260Q binary-iodide and I260Q ternary structures show the expected differences, such as the lack of the incoming nucleotide in the binary complex and movement of the primer terminus as well as several nearby residues (Figure 2D and E).

### Residues in the binary model were found to adopt conformations usually seen in ternary complexes

Many of the residues that typically undergo movements in the transition from binary to ternary in the WT polymerase were found to already be in their ternary positions in the I260Q binary complex (Table 3). Movements of hinge region residues are suggested to drive the closing motion of the fingers domain upon dNTP binding (47). In the I260Q binary complex, all hinge residues are found to exist in their typical ternary positions (Table 3), with the exception of F272, which shifted only to an intermediate position due to the closing of the fingers domain (Figure 2D).

**Table 3.** Comparisons of residue positions in WT and I260Q binary and ternary complexes

WT ternary 2FMS dA:dUMPNPP	I260Q Binary 6BTE dA	I260Q Ternary 6BTF dA:dUMPNPP	E295K 4M9L dA:dCTP
Ternary	Ternary	Ternary	∼Ternary
Ternary	Binary	Ternary	Ternary
Ternary	Ternary	Ternary	Ternary
Ternary	Intermediate	Near ternary	Intermediate
Ternary	Ternary	Ternary	Ternary
Ternary	Near ternary	Ternary	Near binary
Ternary	Intermediate	Ternary	Intermediate
Ternary	Ternary	Ternary	Unique
Ternary	Ternary	Ternary	–

In addition, similar to the ternary structure, Q260 coordinates novel water molecules that interact with R258 and create a bridge between Q260 and R258 that pulls the arginine into an intermediate position between its usual binary and ternary arrangements. Closing of the fingers domain repositions R283 into a ternary position along with Y271. This arrangement puts F272 into an intermediate position between binary and ternary states as the side chain rotation that occurs coincidently with the D192 coordination of the nucleotide binding metal has yet to occur. Many of these premature movements can be traced directly to the introduction of Q260. In the binary complex, the amino group of the Q260 side chain forms hydrogen bonds with the backbone carbonyls of E295 (in the fingers domain) and hinge-adjacent residue Q264, bringing both into their ternary positions (Supplementary Figure S8B). The WT transition from binary to ternary complex also involves movements in the DNA, particularly in the primer terminus and in the templating base (75). In the I260Q binary complex, the templating base has prematurely shifted into its ternary position, while the primer terminus undergoes a smaller shift but remains in a near-binary position. Finally, two additional hinge residues (L194 and Y265) also prematurely adopt their ternary conformations (Supplementary Figure S8B).

### NMR data suggest that I260Q residues in the binary complex adopt a partially closed fingers conformation

**Figure 3.**
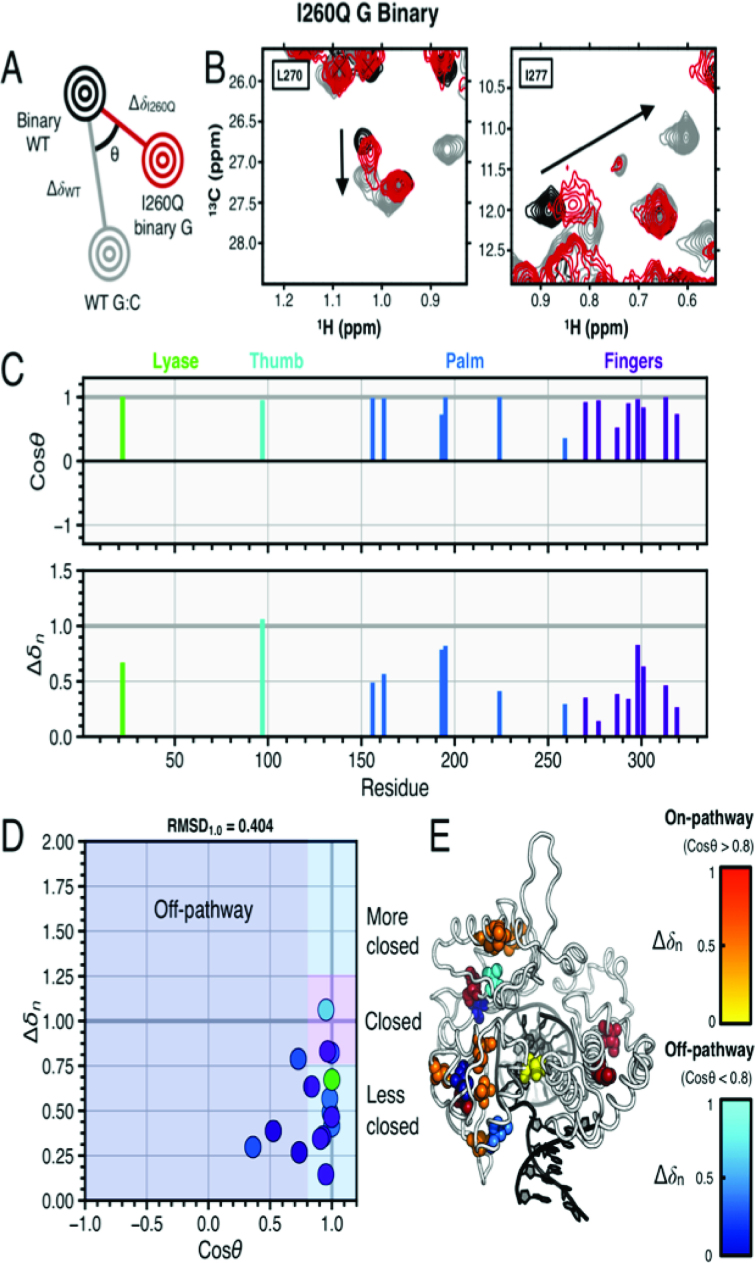
I260Q binary and G:C correct WT ternary complexes. (**A**) Pictorial description of chemical shift vector analysis. (**B**) Experimental NMR data for residues L270 and I277 in WT and I260Q pol β. The spectrum for WT binary (open conformation) is shown in black and WT matched (closed conformation) is shown in gray. The I260Q binary spectrum is shown in red, and the black arrow indicates the direction of the resonance shift upon enzymatic closure. Panel (**C**) shows the cos *θ* and chemical shift magnitude from comparison of the I260Q complex with those of the WT enzyme. The vertical bars are color coded by the lyase, thumb, palm and fingers pol β subdomains. In (**D**), the cos *θ* and Δ*δ_n_* values are shown. These residues in panel (D) are mapped as spheres on the pol β structure shown in (**E**). The spheres shown for residues with cos *θ* > 0.8 (considered on-pathway to closure) are color coded by normalized chemical shift magnitude with red = 0.66–1.0 and off-pathway colored coded in blue.

In all cases described below, NMR-based ligand titrations were consistent with a two-site model. In this analysis, the NMR chemical shifts for open and closed WT pol β are described as a vector with the origin set to the position of the open state resonance (Figure 3A) (76). Any deviations of this same resonance in I260Q from the WT ‘vector’ are analyzed both by magnitude (Δ*δ*_*n*_, Equation 10) and cosine *θ* (Equation 11). If a resonance in I260Q shows identical chemical shifts to WT in both the open and closed conformations, the vectors describing both chemical shifts would be collinear, with cosine *θ* and relative magnitude both equal to 1. I260Q chemical shifts that deviate substantially (cos *θ* < 0.8) would be considered indicative of off-pathway (i.e. non-closure) conformational changes.

To determine if the binary I260Q complex is partially closed, we assessed the conformational states of I260Q by measuring changes in chemical shift magnitude and direction. When considering binary I260Q pol β in relation to the WT binary open and ternary closed spectra with the template G substrate, binary I260Q exhibits distinct resonance shifts from the WT binary and WT ternary closed spectra (Figure 3). Template G binary I260Q pol β appears to adopt or sample an intermediate, semi-closed conformation, as indicated by the 16 major chemical shifts that appear to adopt closed or nearly closed positions as suggested by their chemical shifts (Figure 3C). Notably, there are 12 resonances that correspond to on-pathway chemical shift changes: L22, I97, L156, V162, L195, I224, L270, I277, I298, I293, L301 and V313. Concurrently, there are four resonances that correspond to off-pathway chemical shift changes: V193, L259, L287 and I319. Resonances undergoing both on and off-pathway chemical shift changes can be seen sorted by domain and plotted onto the crystal structure of the ternary enzyme in Figure 3C–E. RMSDs from cos *θ* and Δ*δ_n_* values of 1.0 (RMSD_1.0_, Equation 11) for the fingers and palm subdomains are 0.452 and 0.392, respectively, while the global RMSD_1.0_ across the entire dataset is 0.404, suggesting that the binary enzyme partially closes. Furthermore, analysis of chemical shift perturbations in the I260Q binary complex containing template T DNA compared with those of the WT correct ternary complex (Supplementary Figure S9) also demonstrates that several of the binary resonances are on-pathway state to enzymatic closure, as defined by cos *θ* > 0.8. The residues that experience on-pathway motions to the closed conformation are located in the lyase, palm, thumb and fingers subdomains (Supplementary Figure S9C–E) and demonstrate that the binary template T I260Q enzyme partially closes, similar to the template G complex.

### The I260Q ternary correct is similar to WT ternary correct structure

**Figure 4.**
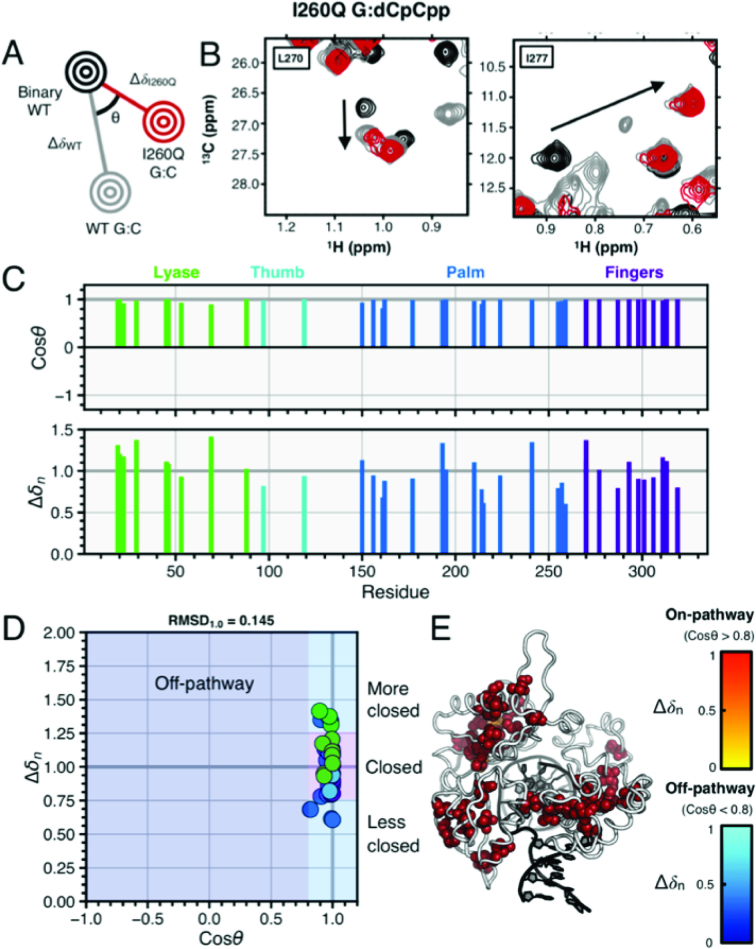
G:C correct I260Q and WT ternary complexes. (**A**) Pictorial description of chemical shift vector analysis. (**B**) Experimental NMR data for residues L270 and I277 in WT and I260Q pol β. The spectrum for WT binary (open conformation) is shown in black and WT correct (closed conformation) is shown in gray. The I260Q G:C spectrum is shown in red, with the black arrow indicating the direction of resonance shifts upon enzymatic closure. Panel (**C**) shows the cos *θ* and chemical shift magnitude from comparison of the two ternary complexes with that of the binary enzyme. The vertical bars are color coded by pol β subdomain. In (**D**), the cos *θ* and Δ*δ_n_* values are shown. These residues in panel (D) are mapped as spheres on the pol β structure shown in (**E**). The spheres shown for residues with cos *θ* > 0.8 (considered on-pathway to closure) are color coded by normalized chemical shift magnitude with red = 0.66–1.0 and off-pathway colored coded in blue.

NMR spectral shifts for the ternary I260Q G:dCpCpp complex (Figure 4 A–D) show marked similarity to the ternary WT complex with a correct nascent base pair, in that it appears to close nearly fully based on comparison with the WT ^1^H/^13^C chemical shifts (Figure 4). Nearly all of the resonances observed are on-pathway to enzymatic closure, as defined by cos *θ* > 0.8. There are 43 resonances exhibiting on-pathway chemical shift changes, corresponding to residues L19, V20, L22, V29, V45, V45, I46, I53, I69, I88, I97, I119, I150, L156, I161, V162, V177, V193, L194, L195, L195, L210, L210, V214, V215, I224, L241, L241, I255, I257, L259, L270, L270, I277, L287, I293, I298, L301, V306, L311, V313, V313 and I319. There are 0 resonances undergoing off-pathway chemical shift changes. Resonances undergoing shifts can be seen plotted by subdomain and on the ternary crystal structure in Figure 4C–E. Individual subdomain RMSD_1.0_ values for the fingers, palm, thumb and lyase subdomains are 0.117, 0.154, 0.096 and 0.164, respectively, while the global RMSD_1.0_ is 0.145 for the entire dataset. Furthermore, similar results are seen in experiments performed with template thymine DNA substrates, as can be seen in Supplementary Figure S10.

### I260Q ternary incorrect is more closed than ternary incorrect WT complex and exhibits more off-pathway changes

**Figure 5.**
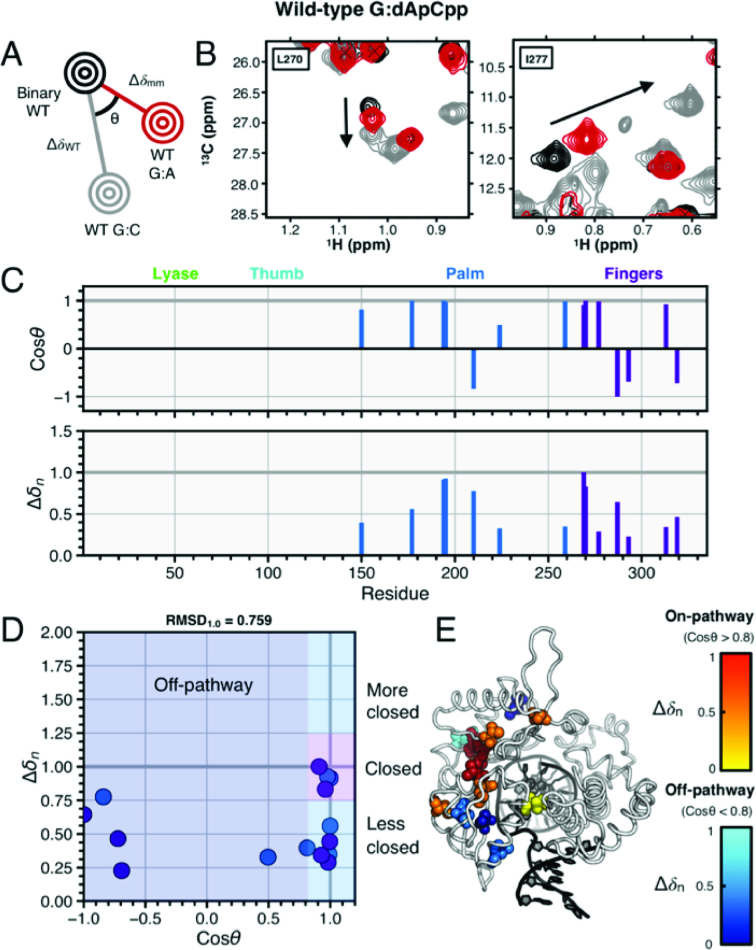
G:A incorrect WT and G:C correct WT ternary complexes. (**A**) Pictorial description of chemical shift vector analysis. (**B**) Experimental NMR data for residues L270 and I277 in WT correct and incorrect pol β. The spectrum for WT binary (open conformation) is shown in black and WT correct (closed conformation) is shown in gray. The WT G:A spectrum is shown in red, with the black arrow indicating the direction of resonance shifts upon enzymatic closure. Panel (**C**) shows the cos *θ* and chemical shift magnitude from comparison of the two ternary complexes with that of the binary enzyme. The vertical bars are color coded by pol β subdomain. In (**D**), the cos *θ* and Δ*δ_n_* values are shown. These residues in panel (D) are mapped as spheres on the pol β structure shown in (**E**). The spheres shown for residues with cos *θ* > 0.8 (considered on-pathway to closure) are color coded by normalized chemical shift magnitude with red = 0.66–1.0 and off-pathway colored coded in blue.

A comparison of correct (G:dCpCpp) WT with incorrect (G:dApCpp) WT ternary complexes was performed to assess the ability of incorrect base pairing to facilitate closure and provide a baseline for comparisons with incorrect I260Q complexes. Chemical shift analysis of the incorrect WT G:dApCpp complex versus WT G:dCpCpp (Figure 5) yields numerous chemical shift changes, with 10 on-pathway resonance shifts (I150, V177, L194, L195, L259, V269, L270, L270, I277 and V313) and 5 off-pathway shifts (L210, I224, L287, I293 and I319). RMSD_1.0_ values for the fingers and palm subdomains are 0.871 and 0.608, respectively, and the global RMSD_1.0_ is 0.759. Therefore, chemical shift perturbations here indicate that the WT incorrect complex does not close, but remains in a conformation distinct from both the closed correct and open binary conformations, as seen previously (77). A distinct T:dCpCpp conformation is also observed in template thymine data, but is difficult to identify due to difficulty in assigning the T:dCpCpp ILV spectrum (Supplementary Figure S11).

**Figure 6.**
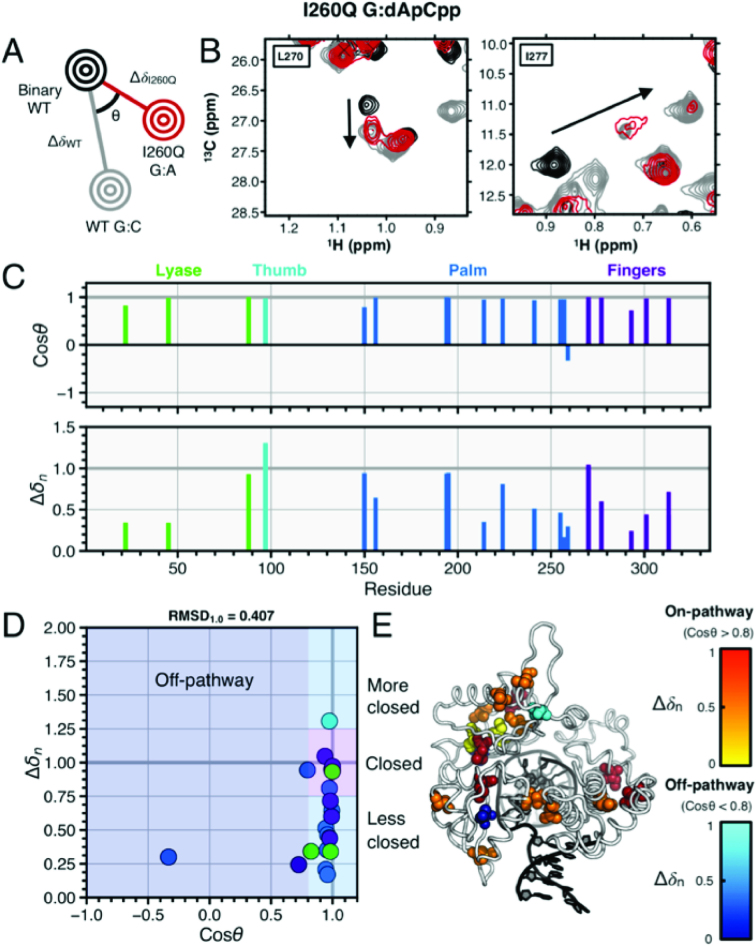
G:A incorrect I260Q and G:A correct WT ternary complexes. (**A**) Pictorial description of chemical shift vector analysis. (**B**) Experimental NMR data for residues L270 and I277 in WT and I260Q pol β. The spectrum for WT binary (open conformation) is shown in black and WT correct (closed conformation) is shown in gray. The I260Q G:A spectrum is shown in red, with the black arrow indicating the direction of resonance shifts upon enzymatic closure. Panel (**C**) shows the cos *θ* and chemical shift magnitude from comparison of the two ternary complexes with that of the binary enzyme. The vertical bars are color coded by pol β subdomain. In (**D**), the cos *θ* and Δ*δ_n_* values are shown. These residues in panel (D) are mapped as spheres on the pol β structure shown in (**E**). The spheres shown for residues with cos *θ* > 0.8 (considered on-pathway to closure) are color coded by normalized chemical shift magnitude with red = 0.66–1.0 and off-pathway colored coded in blue.

In Figure 6, we also show that the incorrect I260Q complex does not closely resemble the WT closed G:dCpCpp correct ternary complex, and by extension does not fully resemble the closed I260Q correct complex. These results further indicate that the I260Q polymerase does not fully close with the incorrect dApCpp, but closes more than the WT incorrect complex, with 19 on-pathway to closure resonances (L22, V45, I88, I97, L156, L194, L195, L195, V214, I224, L241, I255, I257, L270, L270, I277, L301, V313 and V313) and 3 off-pathway resonances (I150, L259 and I293). Residues discussed here can be seen sorted by subdomain and plotted onto the ternary structure in Figure 6C–E. Here, subdomain RMSD_1.0_ values for fingers, palm and lyase subdomains are 0.33, 0.465 and 0.388, respectively, and 0.407 globally. The additional closure in incorrect I260Q compared to incorrect WT may partially explain the reduction in polymerase fidelity in this mutant. Finally, significant chemical shifts are also seen in the I260Q T:dCpCpp incorrect complex as seen in Supplementary Figure S12.

### NMR suggests that the I260Q binary complex resembles the I260Q incorrect ternary complex

When considering the above data alongside each other, it can be seen that the I260Q correct complex has the most chemical shift perturbations of the mutant complexes with 43, with nearly all shifts being clustered around 1 for direction and magnitude. Conversely, the incorrect and binary comparisons yield 22 and 16 significant chemical shift changes, respectively. Notably, average Δ*δ*_*n*_ and cos *θ* values for each comparison suggest that the I260Q correct complex closes similarly to WT, while the I260Q incorrect and binary complexes remain partially closed, with average Δ*δ_n_* values for the I260Q correct, incorrect and binary of 1.012, 0.622 and 0.534, respectively, and average cos *θ* values for I260Q correct, incorrect and binary of 0.974, 0.888 and 0.866, respectively. The number of shifts for each comparison, when combined with the averaged parameters, suggests that the I260Q correct complex closes nearly like WT, while the incorrect and binary complexes remain partially closed, with some off-pathway conformations.

**Figure 7.**
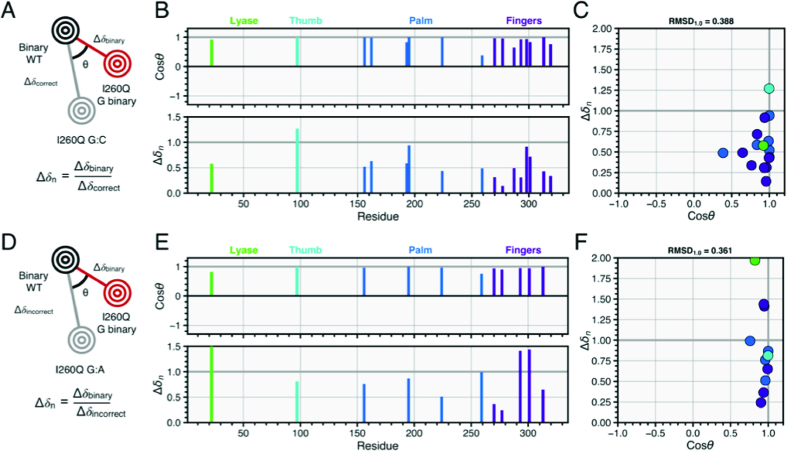
Comparisons between I260Q binary and ternary complexes. (**A**) Vector analysis as described previously, with I260Q binary G compared to the I260Q G:C vector. Panel (**B**) shows the cos *θ* and chemical shift magnitude from comparison of the two ternary complexes with that of the binary enzyme. The vertical bars are color coded by pol β subdomain. In (**C**), the cos *θ* and Δ*δ_n_* values are shown. (**D**) Vector analysis as described previously, with I260Q binary G compared to the I260Q G:C vector. Panel (**E**) shows the cos *θ* and chemical shift magnitude from comparison of the two ternary complexes with that of the binary enzyme. The vertical bars are color coded by pol β subdomain. In (**F**), the cos *θ* and Δ*δ_n_* values are shown.

We wondered if the higher affinity of I260Q for incorrect dNTP and its partially closed binary form indicated that perhaps I260Q in its binary form was similar to its ternary conformation in the presence of the incorrect dNTP. Therefore, we compared the I260Q binary NMR spectrum to the NMR spectrum for the ternary correct and incorrect I260Q complexes (Figure 7). These comparisons show that the binary I260Q resonances more closely resemble the incorrect I260Q ternary resonances, with RMSD_1.0_ values of 0.388 and 0.361 for the correct and incorrect comparisons, respectively. Moreover, as shown in Figure 7F, chemical shift vectors for the I260Q incorrect and binary complexes resemble each other from the perspective of their similar Δ*δ_n_* and cos *θ* values, and the similar RMSD values can be explained by several residues in the I260Q G binary–I260Q G:dApCpp comparison that have high Δ*δ_n_* values but maintain cos *θ* values close to 1. Taken together, these results suggest that the I260Q mutator binary complex is in a conformation that is already poised for interactions with the incorrect dNTP. The fact that the binary I260Q complex is more similar to the incorrect ternary complex helps explain the higher affinity for incorrect dNTP binding and its inability to properly discriminate correct from incorrect dNTP substrate during ground state binding. This conclusion is less clear in template thymine work as shown in the Supplementary Figure S13, with RMSD values between correct and incorrect comparisons (0.366 and 0.381, respectively) more similar to each other. This similarity in RMSD values is likely due to the similarity between the correct and incorrect conformations seen in Supplementary Figure S11.

^1^H-^13^C methyl ILV CPMG relaxation dispersion data indicate millisecond motions in proteins. In this work, to gauge general flexibility and dynamics in WT and I260Q enzymes, CPMG experiments were conducted. These data show 37 residues in the WT G:dApCpp incorrect ternary complex undergoing millisecond motions, indicating increased flexibility in the incorrect G:dApCpp WT complex compared to 14 flexible residues in the correctly paired G:dCpCpp WT complex (Supplementary Figure S14). These data indicate that the WT incorrect conformation remains in a state of increased flexibility. Furthermore, CPMG relaxation dispersion analysis yields 18 residues with millisecond motions for the I260Q correct G:dCpCpp complex and 15 for the I260Q G:dApCpp incorrect complex, indicating potentially impaired flexibility in the I260Q incorrect G:dApCpp complex (Supplementary Figure S14). Moreover, in template thymine complexes, 8 and 15 flexible residues are observed for I260Q correct and incorrect complexes, respectively (Supplementary Figure S14). The number of flexible residues in the incorrect I260Q complex is reduced when compared to the incorrect WT complex, but the significance of flexibility changes is unclear. Nonetheless, the ability of pol β to access the correct conformations indicates an innate flexibility, which is altered in the I260Q mutant. This observation is consistent with prior work from our group (77). Due to ambiguity in conclusions from CPMG data described here, it is most likely that altered binary ground state binding conformations in the I260Q complexes explain altered incorrect dNTP binding.

### Stopped-flow fluorescence reveals an altered catalytic scheme for I260Q

Previous work from our laboratory has shown that precatalytic conformational changes of pol β play a key role in substrate specificity (25). Pol β first binds single-nucleotide gapped DNA (DNA*_n_*) to form the binary complex. The binary complex binds dNTP resulting in movement of the fingers to a more closed conformation (β’). Following fingers closing, a non-covalent step occurs that is dependent on the catalytic metal and the 3′OH of the primer terminus (25). Next, the nucleotidyl transfer reaction is carried out to add the dNTP to the single-nucleotide gapped DNA, forming nicked DNA (DNA_+1_) and pyrophosphate (PP_i_). Finally, pol β opens (39), and the bound PPi is released followed by DNA product release.

To measure the rates of precatalytic conformational changes upon nucleotide binding, stopped-flow experiments were performed under single turnover conditions with extendable DNA and correct dCTP. We observe an initial quenching of fluorescence associated with fingers domain closure followed by a gradual recovery of fluorescence as the fingers domain re-opens (Supplementary Figure S15). We also directly measured reverse closing rates for WT and I260Q as previously described (59) by mixing a solution containing a pol β-dideoxyG-correct dCTP ternary complex and 10 mM MgCl_2_ with a 10-fold excess of an unlabeled pol β-extG binary complex. The unlabeled enzyme: DNA complex rapidly traps the released dCTP. The fluorescence traces of the reverse closing for both WT and I260Q are shown in Supplementary Figure S15. The traces for the reverse steps for both WT and I260Q fit best to a triple-exponential function that we modeled as follows:

\begin{eqnarray*}{\rm{\beta ^{\prime \prime}}} \vdots {\rm{DN}}{{\rm{A}}_n} \vdots {\rm{dNTP}}\mathop \to \limits^{\mathop {{k_{ - 4}}}\limits^{(1)} } {\rm{\beta ^\prime}} \vdots {\rm{DN}}{{\rm{A}}_n} \vdots {\rm{dNTP}}\mathop \to \limits^{\mathop {{k_{ - 3}}}\limits^{(2)} } {\rm{\beta }} \vdots {\rm{DN}}{{\rm{A}}_n} \vdots {\rm{dNTP}}\mathop \leftrightarrow \limits^{\mathop {{K_{2}}}\limits^{(3)} } {\rm{\beta }} \vdots {\rm{DN}}{{\rm{A}}_n}\end{eqnarray*}

**Figure 8.**
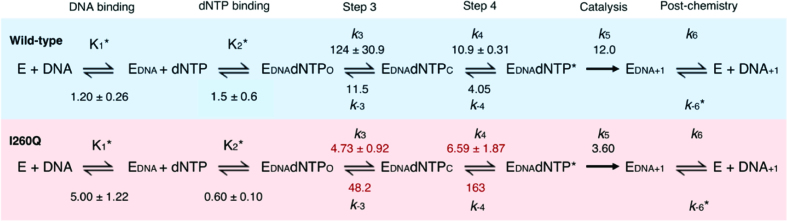
Catalytic scheme with fixed reverse rates modeled in KinTek Global Explorer for template G. All units shown here are s^−1^, except for those marked by *, which have units of nM^−1^ and μM^−1^ for the DNA and dNTP binding steps, respectively. *k_-6_* steps have units of μM^−1^s^−1^.

Here, step (1) is defined as the reverse non-covalent step, *k_-4_*, step (2) is the reverse of fingers closing, *k_-3_*, and step (3) is the release of the dNTP. We constrained the dNTP binding ratio *K*_2_ to the *K*_d(dNTP)_ obtained from single turnover experiments. The best-fit rates we obtained via KinTek Global Explorer are included in Figure 8.

The forward rate (*k*_+3_), usually referred to as fingers closing, is faster for WT (124 ± 30.9 s^−1^) than for I260Q (4.73 ± 0.92 s^−1^) and the reverse rate (*k*_-3_) for I260Q (48.2 s^−1^) is three times faster than WT (11.5 s^−1^). This suggests that fingers closing occurs for WT but not for I260Q pol β. The forward rates of the second non-covalent step (*k*_+4_) for WT and I260Q are similar. However, the reverse rate of this step (*k*_-4_) is much faster for I260Q (163 s^−1^) than for WT pol β (4.05 s^−1^). Therefore, the forward and overall rates of the precatalytic conformational changes we are able to detect with this methodology are significantly slower for I260Q than for WT pol β. Modeling of the stopped-flow kinetic traces for incorporation of dATP opposite template T yielded similar results (Supplementary Figure S15). From these observations, it is evident that I260Q exhibits markedly altered equilibria of closure of the fingers subdomain and non-covalent steps.

Identical stopped-flow FRET experiments with incorrect dATP opposite template G (Supplementary Figure S16) or dCTP opposite template T (Supplementary Figure S16) did not yield any noticeable fluorescence change over the 10 s time course examined for WT and I260Q. The reasons for this are unclear, but may be due to the difficulty of detecting incorrectly paired complexes of pol β as noted in previous studies (25,59).

## DISCUSSION

The faithful incorporation of nucleotides by DNA polymerases has long been appreciated in terms of proper hydrogen bonding networks between the protein and substrate in combination with those from the Watson–Crick pairing. More recently, a growing body of evidence has implicated that structural dynamics between discrete conformational states of the polymerase play significant roles in nucleotide selection (26–29,40,42,78,79). A finer understanding of the control of these dynamics will provide insight into the mechanistic basis of DNA mutation and highlight potential pathways of cancer biogenesis. Previous molecular modeling studies have suggested that the rate-limiting step that prevents incorporation of the incorrect dNTP in WT pol β is not a chemical step, but rather one or more conformational rearrangements that occur in residues that bring the enzyme into an active state upon binding dNTP (80). Previous structural and FRET studies have also suggested that the fidelity of DNA pol β is governed by a combination of global conformational changes (fingers domain opening and closing) and minor local rearrangements during nucleotide selection (20,25). In this study, we employed a combination of stopped-flow FRET, X-ray crystallography and NMR to characterize the dynamics and conformational landscape of the I260Q pol β mutator variant. Our results demonstrate that the I260Q low fidelity variant of DNA pol β exhibits reduced conformational sampling and elimination of an ‘open’ binary conformational state. We suggest that the elimination of this binary ‘open’ conformational state typically involved in catalysis provides a gateway to error incorporation and may facilitate tight binding of the incorrect dNTP by I260Q.

### I260Q pol β exhibits a partially closed binary structure

First, we examined the DNA*_n_*-bound WT binary complex, as it is poised to discriminate between correct and incorrect dNTPs. FRET data indicate that the distance between the fluorophore and quencher is ∼43.2 Å with guanine in the gap, and 43.9 Å in WT pol β with thymine in the gap. These values are in agreement with X-ray structural data for the binary complex, with a calculated distance of 43.8 Å (17) from the alpha carbon of V303 to the DNA residue 8 positions upstream in binary structure 1BPX. These data indicate that the WT binary enzyme forms an open structure that is consistent with known crystallography data, in which helix N and M are not yet fully closed around the DNA and nucleotide substrate.

In contrast, the binary complex of the mutator I260Q pol β exists in a partially closed conformation. The calculated FRET distance between the donor/acceptor pair is ∼37.1 Å with template guanine and 39.8 Å with template thymine, 6 and 4 Å closer than in the two respective WT pol β binary complexes. These data are supported by NMR chemical shift analyses that show I260Q binary resonances located at intermediate values between open and fully closed conformations (Figure 3). Mutation of I260 facilitates partial enzyme closure in the absence of dNTP substrate, which could potentially explain the tighter binding of incorrect nucleotide to I260Q (*K*_d_ = 16 μM) versus that for WT (*K*_d_ = 365 μM).

We also demonstrate that the crystal structure of pol β I260Q assumes a closed fingers conformation in the absence of incoming nucleotide. In the binary I260Q variant, many residues were found to be in positions analogous to those of the WT ternary structure, indicating that several of the conformational movements of amino acid residues of I260Q pol β that allow for proper selection may not be able to occur prior to nucleotide binding. Several of the prematurely closed residues have been implicated as being important to polymerase fidelity, including hinge region residue Y265 (81) and fingers residue R283 (75), which interacts with the templating base to bring it to its proper position to coordinate an incoming nucleotide. Therefore, distances calculated from the steady-state FRET and the crystal structure support the presence of a closed binary state, which suggests that the ground state for nucleotide binding for I260Q is partially closed and that this conformation renders the enzyme defective in nucleotide discrimination. Therefore, we conclude that the precise conformational movements that occur upon the binding of dNTP to the binary complex of pol β are critical for substrate selection. Alteration of the I260 hinge residue likely renders the enzyme impaired in its ability to sample the proper conformational landscape, resulting in its loss of fidelity. We speculate that the incorrect nucleotide could slide into the closed active site with minor adjustments.

Given the ∼35-fold lack of nucleotide discrimination in the ground state binding step of the I260Q mutant, our observations give rise to several conclusions. The fact that the binary state appears to be sampling a partially closed or closed form indicates that ground state binding itself has been disrupted and that it may be in transition to the incorrect dNTP-bound conformation even without the nucleotide present, as suggested by Figure 7. This marks a striking departure from the behavior of the WT enzyme, which remains in a largely open state in the binary complex. It should be noted that minor chemical shift changes in the fingers subdomain of I260Q are observed in the apoenzyme (Supplementary Figure S3), an observation that suggests that equilibrium between open and closed states of the subdomain is perturbed even with no DNA bound.

### I260Q and wild-type incorrect complexes adopt similar conformational states

Study of the incorrect complexes of the mutant and WT enzymes indicates that their NMR spectra are similar. Strikingly, the incorrect conformations formed in the guanine and thymine ternary complexes are similar between the I260Q and WT enzymes for each respective incorrect base pair, suggesting that the I260Q mutation stabilizes a WT-like incorrect state, as opposed to a more unique conformation. This observation highlights the importance of the polymerase forming the closed ternary conformation for incorporation of nucleotides and suggests that incorrect complexes fail to close completely around the nucleotide.

### I260Q exhibits altered conformational dynamics

Current and previous measurements of the rates of conformational changes of WT pol β with various DNA primer-templates (25,59) reveal the presence of at least two non-covalent steps preceding the chemical reaction. The first of these steps has a fast forward rate and is assumed to represent the closing of the fingers domain upon the binding of the correct dNTP (20). The second movement is slow and represents a metal-dependent non-covalent step (25). Similar results have been observed for the Klenow fragment of DNA polymerase I (37). These non-covalent steps are also present during the reaction pathway of I260Q (Figure 8). However, the measured rates of the reverse reactions of I260Q using a binary complex as a trap indicate that both fingers closing and the metal-dependent second non-covalent steps favor the reverse reaction and that both of these steps have slower forward rates than WT pol β. This can also be seen from the shallow fluorescence changes in the I260Q FRET traces in Supplementary Figure S15 compared to the larger changes in fluorescence observed for WT, which indicate larger domain movements. We obtained a fast closing rate of *k*_+3_ for WT in the presence of correct nucleotide, at 124 s^−1^. In contrast, the opening rate for the WT ternary complex (*k*_-3_) is significantly slower at 11.5 s^−1^, heavily biasing the equilibrium toward the closed state upon binding of the correct dNTP. These two rates ensure that after nucleotide binding and fingers-closing, nucleotide incorporation is overwhelmingly likely to occur, since the rate of fingers-opening is similar to the rate-limiting step in the forward direction, based on single turnover nucleotide incorporation rates. This large overall rate difference results in fidelity being less dependent on the exact rate constant of the rate-limiting step. This fidelity feature was also shown for both T7 DNA polymerases (43) and KF (82), which also exhibit a rapid conformational change upon binding of a correct nucleotide; the slow reversal of this change delays nucleotide release, biasing the reaction toward the productive forward direction. For I260Q, the slower forward rates may be explained by the location of I260 in the hydrophobic hinge, and an overall impairment in the ability of I260Q to conformationally rearrange compared to WT enzyme. The faster reverse rates for I260Q suggest that the partially closed I260Q binary structure is stable and that I260Q prefers to populate this state. The stability of this binary state may be due to the newly formed hydrogen bonds between Q260 and its neighboring E295 and Q264 residues (Supplementary Figure S8). However, if and when I260Q does manage to proceed through the two non-rate-limiting conformational changes, it can perform chemistry and insert the nucleotide into the single base pair gap, albeit at a *k*_pol_ that is three times slower than WT. This slower rate of polymerization may be indicative of a smaller population of ternary complexes making it to the chemistry step, or perhaps results from impairment of the ability of I260Q to adjust to correct and incorrect nascent base pairs in a conformational manner.

Taken together, our results suggest that conformational dynamics important for substrate discrimination are not able to take place in I260Q because of its partially closed state in binary form. Since the I260Q mutator fails to transition efficiently from the partially closed binary conformation to the closed conformation, our results indicate that the initial binary conformation assumed before dNTP binding is important for fidelity and that the I260 residue is essential for the discrimination between complementary and non-complementary nucleotides. Our results support previous hypotheses (46) that suggest a hindered dNTP closing for this enzyme accounts for its altered fidelity and disagree with previous studies that show both WT and I260Q possess fast conformational closing steps during both correct and incorrect dNTP incorporation (83).

### WT and ternary I260Q complexes highlight multiple pathways for incorrect incorporation

Using NMR, the correct ternary G:dCpCpp I260Q complex (Figure 4) shows spectra quite similar to the WT correct ternary complex (vector RMSD_1.0_ = 0.145), indicating near-WT levels of closure. This result highlights the ability of the nucleotide selection subdomain to close with the mutation in the hinge, confirming that the mutant polymerase can populate catalytically relevant conformations and proceed through chemistry.

In contrast, both the WT incorrect (Figure 4) and I260Q incorrect (Figure 5) G:dApCpp complexes fail to close as well as the correct ternary complex, with I260Q closing more than WT. Furthermore, Figure 7 also shows that the I260Q binary form of the mutator most resembles the I260Q incorrect form and not the I260Q correct ternary complex. This is an intriguing observation and is an idea debated in much previous work (40,41,83), whether correct and incorrect dNTP complex formation and incorporation proceed through the same or different kinetic conformations and therefore different mechanisms of incorporation. We suggest that correct and incorrect nucleotide incorporation of different dNTPs occur via different pathways. Furthermore, the increased flexibility of WT incorrect complexes using CPMG relaxation data (Supplementary Figure S14) when compared to incorrect complexes of the I260Q mutator demonstrates a potentially important role for enzyme flexibility in nucleotide selection.

### Rethinking models of nucleotide binding

**Figure 9.**
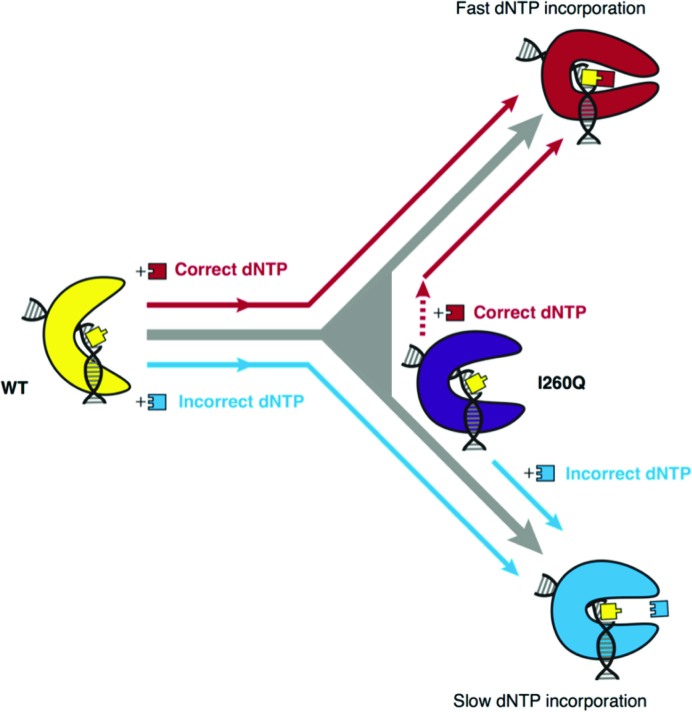
Cartoon representation of pol β’s nucleotide selection mechanism. Binary I260Q is observed to be in an intermediate conformation, reverting to a WT closed-like conformation with a correct nucleotide and a WT open-like conformation with an incorrect nucleotide.

The fact that the ground state binding conformation of the I260Q mutant impacts binding so drastically in large part suggests that the mutant has an impaired ability to assess accuracy of bound nucleotides and is poised to bind incorrect dNTPs while retaining its ability to enter catalytically competent conformations with correct dNTPs. Moreover, the hydrophobic packing of I260 within the hydrophobic hinge most likely precludes the WT enzyme from entering stable incorrect states. Most notably, this observation highlights the necessity in the WT of conformational ‘checking’ of accuracy of bound dNTPs based on the templating residue. A pictorial representation of the conformational and catalytic landscape of pol β can be seen in Figure 9. In the WT enzyme, it is critical that the polymerase retain the ability to populate native open binary, closed correct ternary and alternate incorrect ternary conformations, with any perturbation to this conformational landscape impairing enzymatic fidelity. The binary complex can be considered to bind and gauge the accuracy of dNTPs, and subsequently trigger corresponding conformational changes. In the case of matched nucleotides, it responds by entering the catalytically competent state. However, in the case of incorrect nucleotides, it enters a distinct conformation that deters catalysis and allows the incorrect dNTP to dissociate. However, in the case of I260Q, this binary conformation's fundamental ability to assess nucleotide accuracy is disrupted that results in binding of all nucleotides, correct or incorrect. Moreover, it is likely that I260Q forms incorrect conformations more easily than the WT, given that the binary complex exhibits some characteristics of the incorrect complex with no nucleotide at all. This observation emphasizes the importance of precatalytic motions and conformational changes to enzymatic fidelity and stresses the critical role that a native conformational landscape plays in pol β’s ability to faithfully repair DNA. Moreover, the work presented here not only highlights the nature of accuracy assessment by the precatalytic ternary complex of pol β, but also the role of the hydrophobic hinge and protein flexibility in the nucleotide discrimination step of pol β’s catalytic scheme.

## DATA AVAILABILITY

**PDB IDs:** 6BTE and 6BTF. **BMRB IDs:** 27559, 27560 and 27561.

## Supplementary Material

Supplementary Data
