# Supplementary material for: I260Q DNA polymerase β highlights precatalytic conformational rearrangements critical for fidelity
Source: Nucleic Acids Res. 2018 Sep 17;46(20):10740–56. doi: 10.1093/nar/gky825 (PMC6237750; doi:10.1093/nar/gky825)
Supplement: Supplementary Data [file gky825_supplemental_files.docx]

**Supplementary Data**

**Table S1.** Crystallographic data collection and refinement statistics

|  | Ternary dUMPNPP | Binary  Sodium Acetate + Iodide |
| --- | --- | --- |
| PDB ID  Space Group  Cell Parameters (Å) | 6BTF  P2(1)  50.6, 79.9, 55.4  β = 107.4^°^ | 6BTE  P2(1)  50.9, 80.6, 55.1  β = 107.9^°^ |
| Resolution (Å)^a^ | 38 – 1.75  (1.81 – 1.75) | 20 – 2.20  (2.28 – 2.20) |
| Completeness (%) | 99.2 (99.8) | 98.9 (89.3) |
| Unique Reflections  Redundancy | 42075  3.6 (3.3) | 20350  5.6 (2.4) |
| R_meas_ % | 9.8 (53.9) | 7.9 (32.7) |
| R_pim_ % | 5.3 (30.1) | 3.3 (19.4) |
| CC_1/2_ | 0.992 (0.524) | 0.997 (0.884) |
| I/σI | 9.8 (2.0) | 20.4 (3.7) |
| Wilson B-factor (Å^2^**)** | 22.8 | 15.7 |
| Cross R I/F (%) ^b^ | [1] 43.0/25.8  [2] 73.2/44.2  [3] 99.6/57.0  [4] ---/--- | [1] 52.5/31.7  [2] 73.3/44.2  [3] 99.7/55.7  [4] 39.1/25.3 |
| R_work_/R_free_ (%) | 17.6/21.3 | 18.5/22.9 |
| r.m.s.d. bonds (Å) | 0.006 | 0.002 |
| r.m.s.d. angles (°) | 0.963 | 0.4648 |
| Ramachandran Favored  Outlier | 99 %  0 % | 98.1 %  0 % |
| CC model-map | 0.91 | 0.91 |
| B-factors Å^2^ (atoms)  Protein  DNA  Ions  Ligand  Water | 28.0 (2471)  38.6 (596)  32.3 (4)  24.6 (28)  39.7 (338) | 16.5 (2573)  25.3 (631)  27.1 (5)  24.2 (427) |

^a^Numbers in parentheses denote high resolution bin

^b^Cross R against: [1] WT ternary (2FMS*^19^*) [2] WT binary (3ISB*^56^*) [3] E295K dA:dCTP (4M9L*^44^*) [4] I260Q ternary (this study)

**
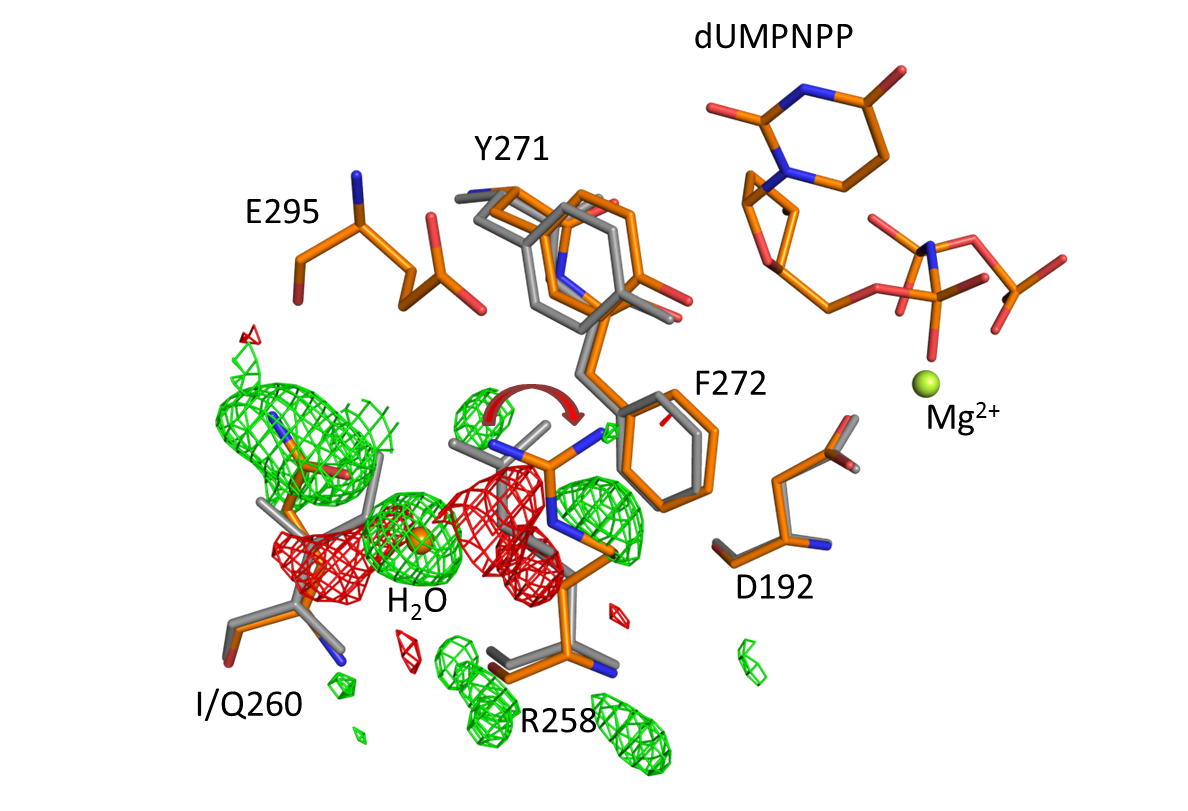
**

**Figure S1.** Superposition of I260Q and WT ternary complexes in the region surrounding the mutation and the incoming nucleotide. The WT ternary (light gray, PDB ID 2FMS*^19^*) and I260Q ternary (orange) structures are superimposed. The isomorphous difference Fourier (Fo _I260Q_-Fo _2FMS_) map between the two datasets is contoured at 3σ (green)/- 3σ (red). The map shows clear density indicating the presence of the mutation and reveals a novel water molecule coordinated by the mutated residue. In the variant structure R258 is seen slightly shifted from its usual conformation in a ternary complex.


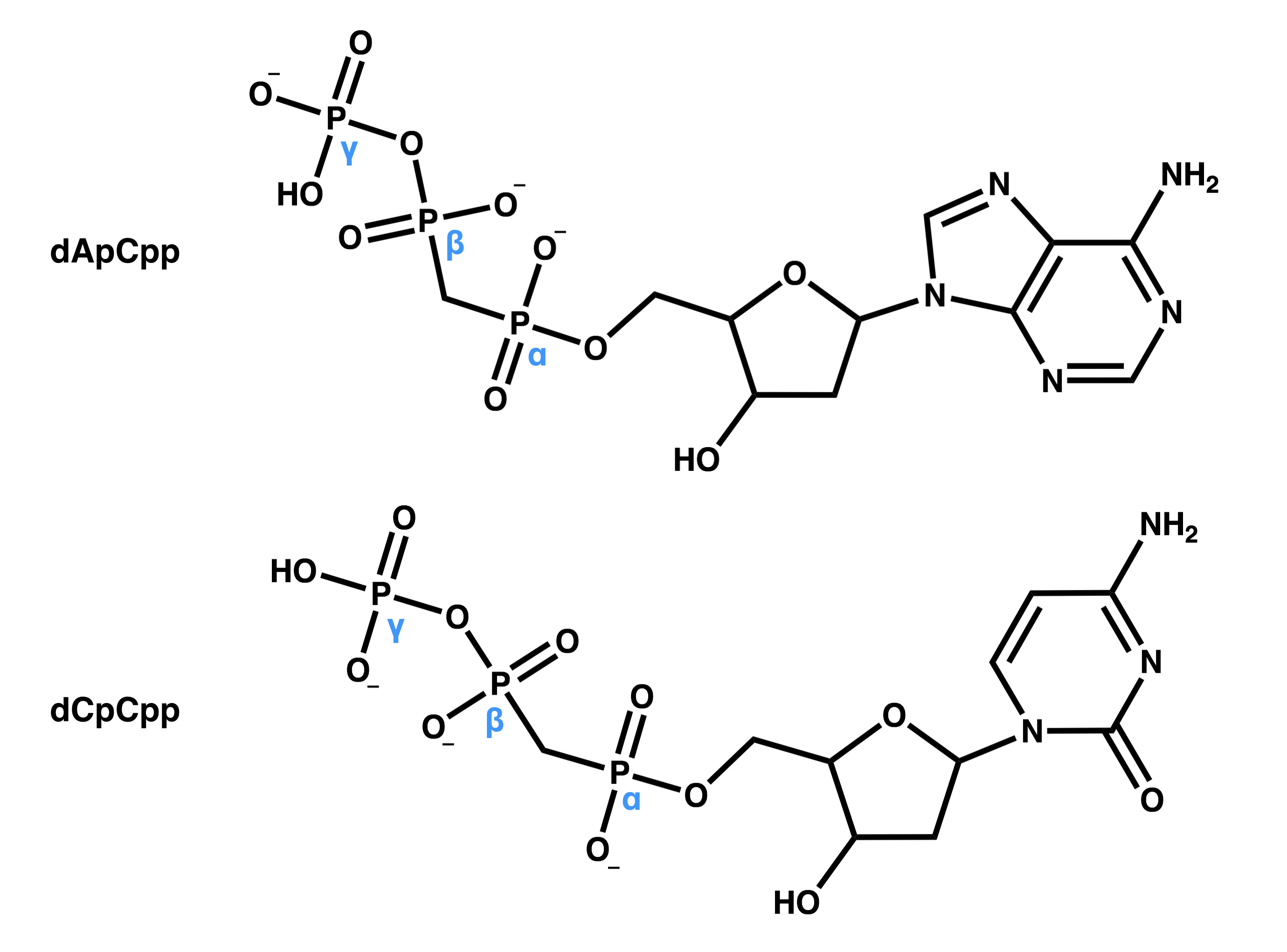
**Figure S2.** Non-hydrolyzable dApCpp and dCpCpp nucleotide analogs used in this work. Here, there are methylene groups in between the α and β phosphates.


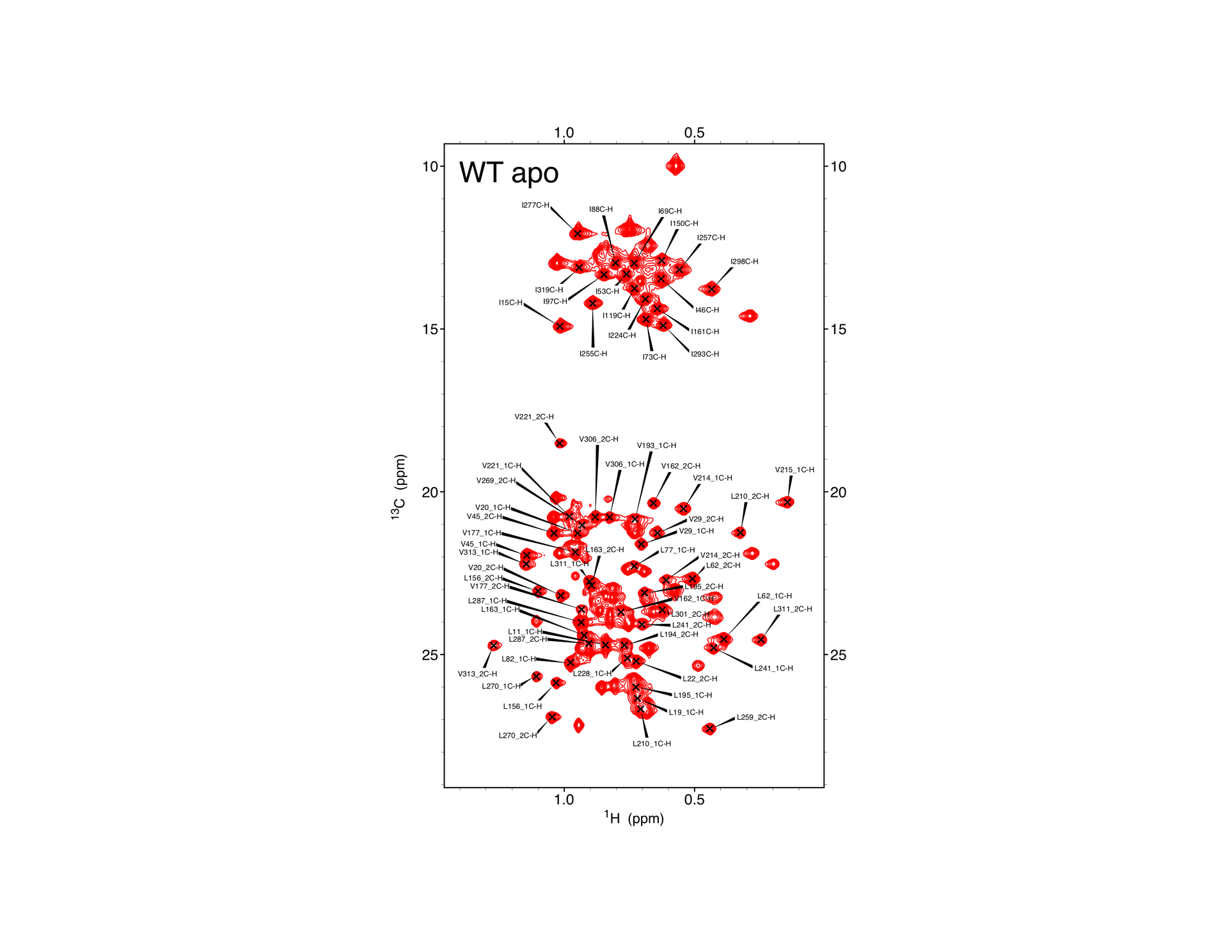


**Figure S3.** Methyl ILV spectra of WT apo pol β. Assigned resonances are shown.


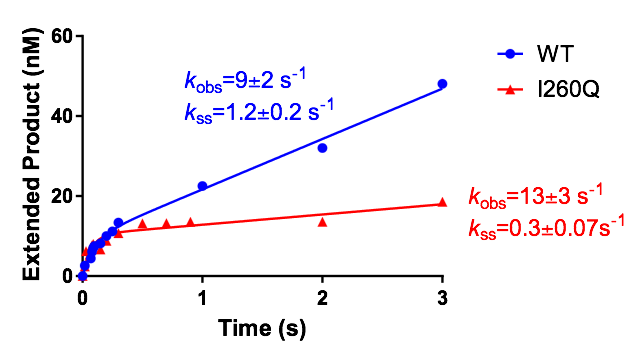


**Figure S4**. **WT and I260Q display pre-steady-state burst activity.** Representative plots are displayed for template G. The biphasic nature of the pre-steady state activity of WT and I260Q on extG DNA are evident in plotted data. Curves show the best fit to Equation 1 for each data set. The two bursts shown here are representative of at least eight replicates, but the parameters of the specific fits that were chosen are shown.

**
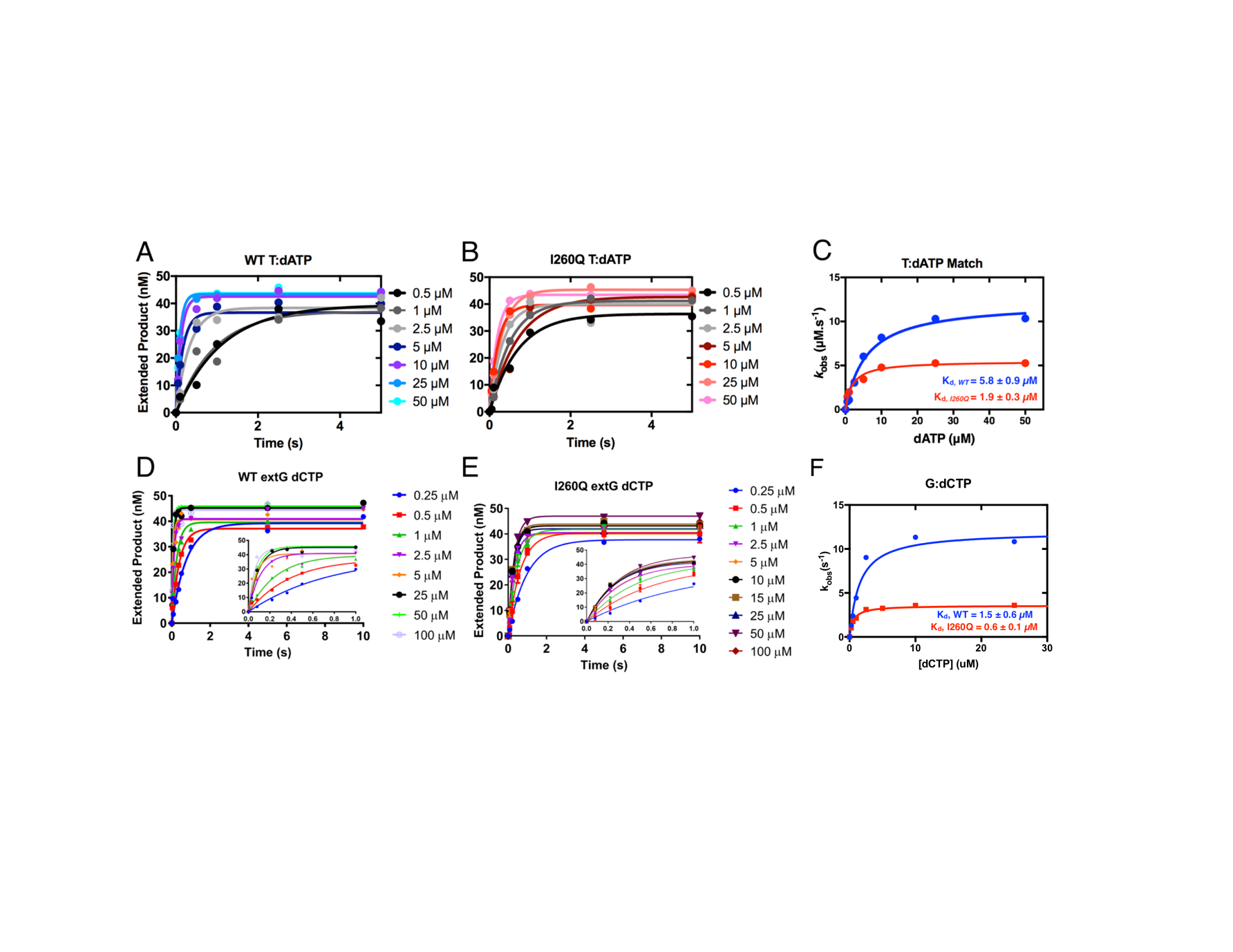
**

**Figure S5.** Product vs. time polymerization single turnover plots fit to equation 2 for WT and I260Q. WT (A) and I260Q (B) pol β with radiolabeled extT and correct dATP were allowed to react with the given dNTP for various amounts of time before the reaction was quenched with 0.5 M EDTA. Figures (D) and (E) are WT and I260Q pol β with radiolabeled extG and correct dCTP. These experiments were performed for a series of dNTP concentrations, and the *k*_obs_ values obtained were used in Equation 3 to calculate the *k*_pol_ and K_d(dNTP)_ values (C/F) presented in Table 2. Insets allow a better view of the fits at the earliest time points. Each data point is an average of up to 3 replicates obtained independently.


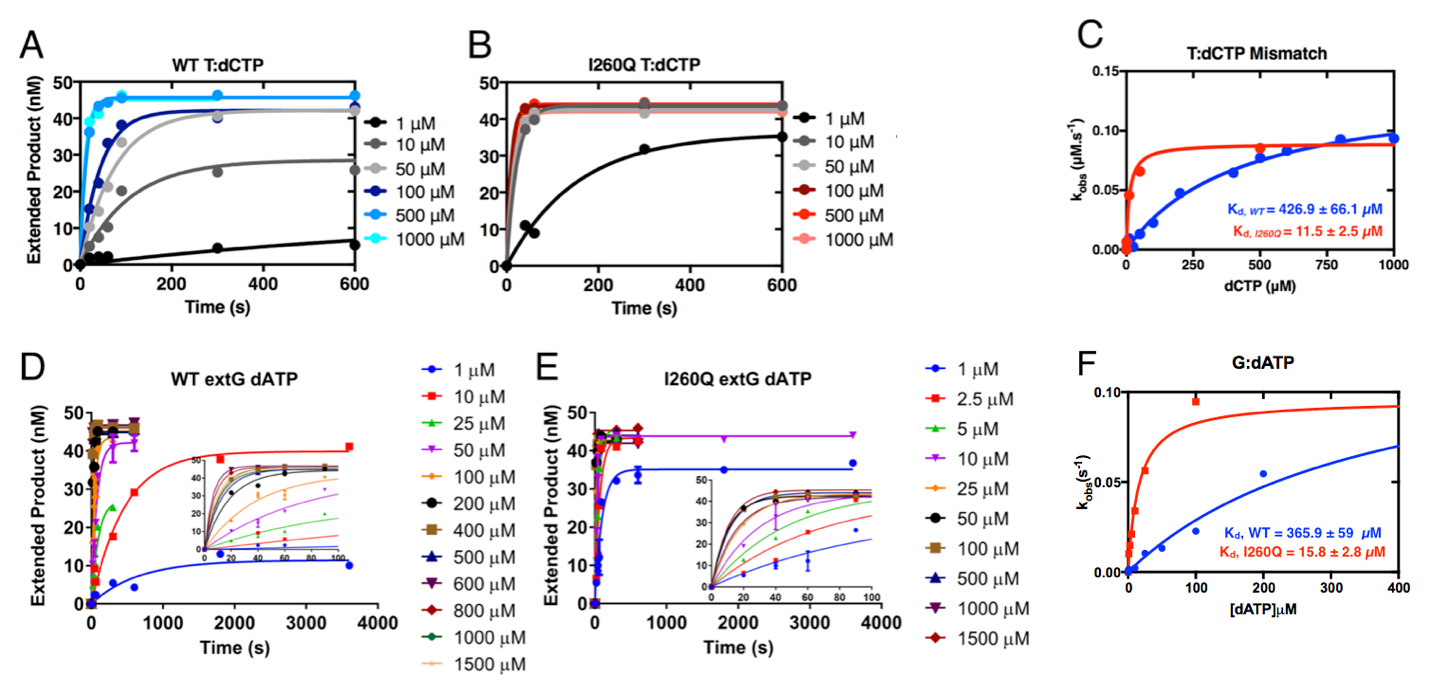


**Figure S6.** Product vs. time polymerization single turnover plots fit to equation 2 for WT and I260Q. WT (A) and I260Q (B) pol β with radiolabeled extT and incorrect dCTP were allowed to react with the given dNTP for various amounts of time before the reaction was quenched with 0.5 M EDTA. Figures (D) and (E) are WT and I260Q pol β with radiolabeled extG and incorrect dATP. These experiments were performed for a series of dNTP concentrations, and the *k*_obs_ values obtained were used in Equation 3 to calculate the *k*_pol_ and K_d(dNTP)_ values (C/F) presented in Table 2. Insets allow a better view of the fits at the earliest time points. All experiments were repeated at two to three times.

**
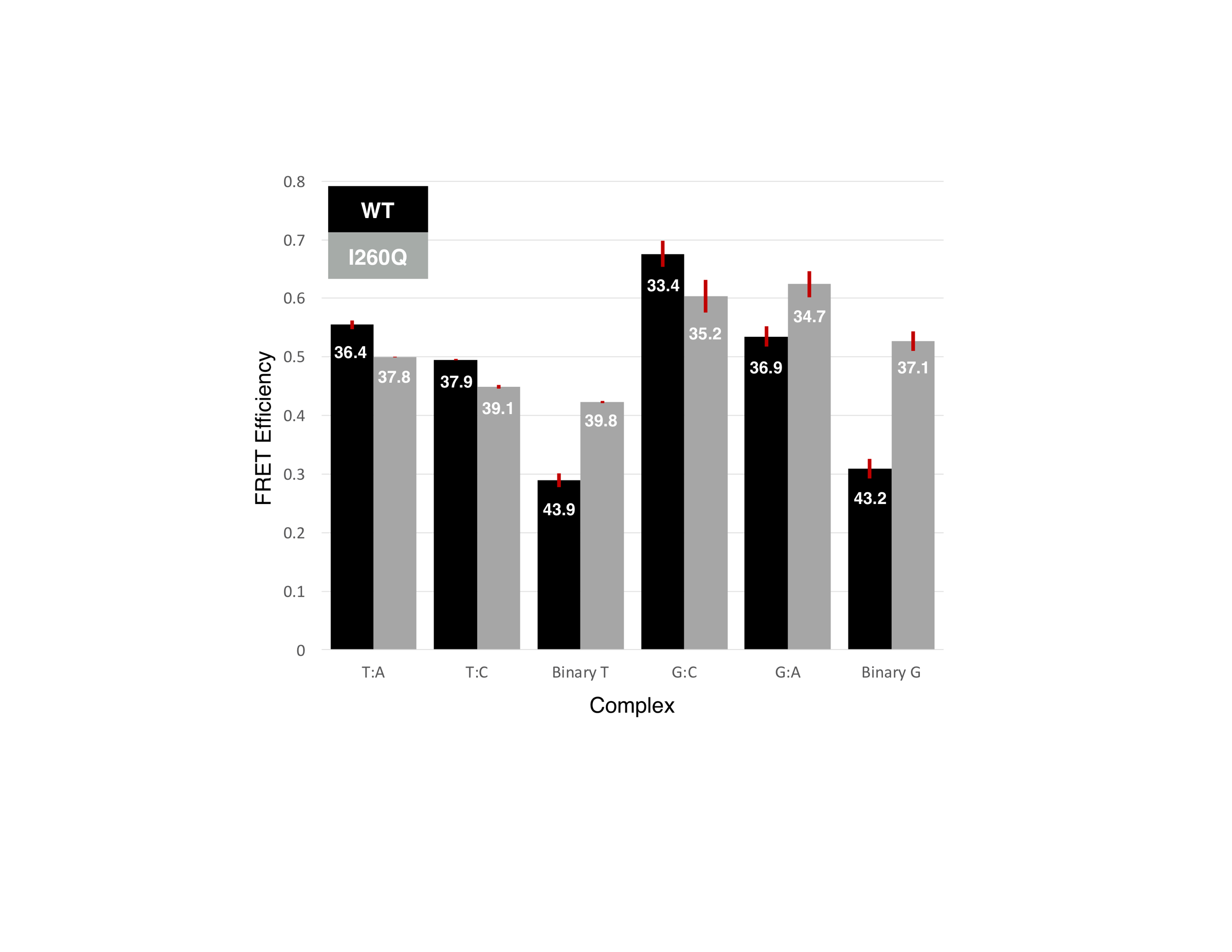
**

**Figure S7. FRET efficiencies and distances calculated from steady-state FRET experiments.** Here, efficiencies from duplicate measurements are plotted as a function of the WT or I260Q complex examined, with error bars shown in red. Calculated distances are provided on each bar in Å.

**
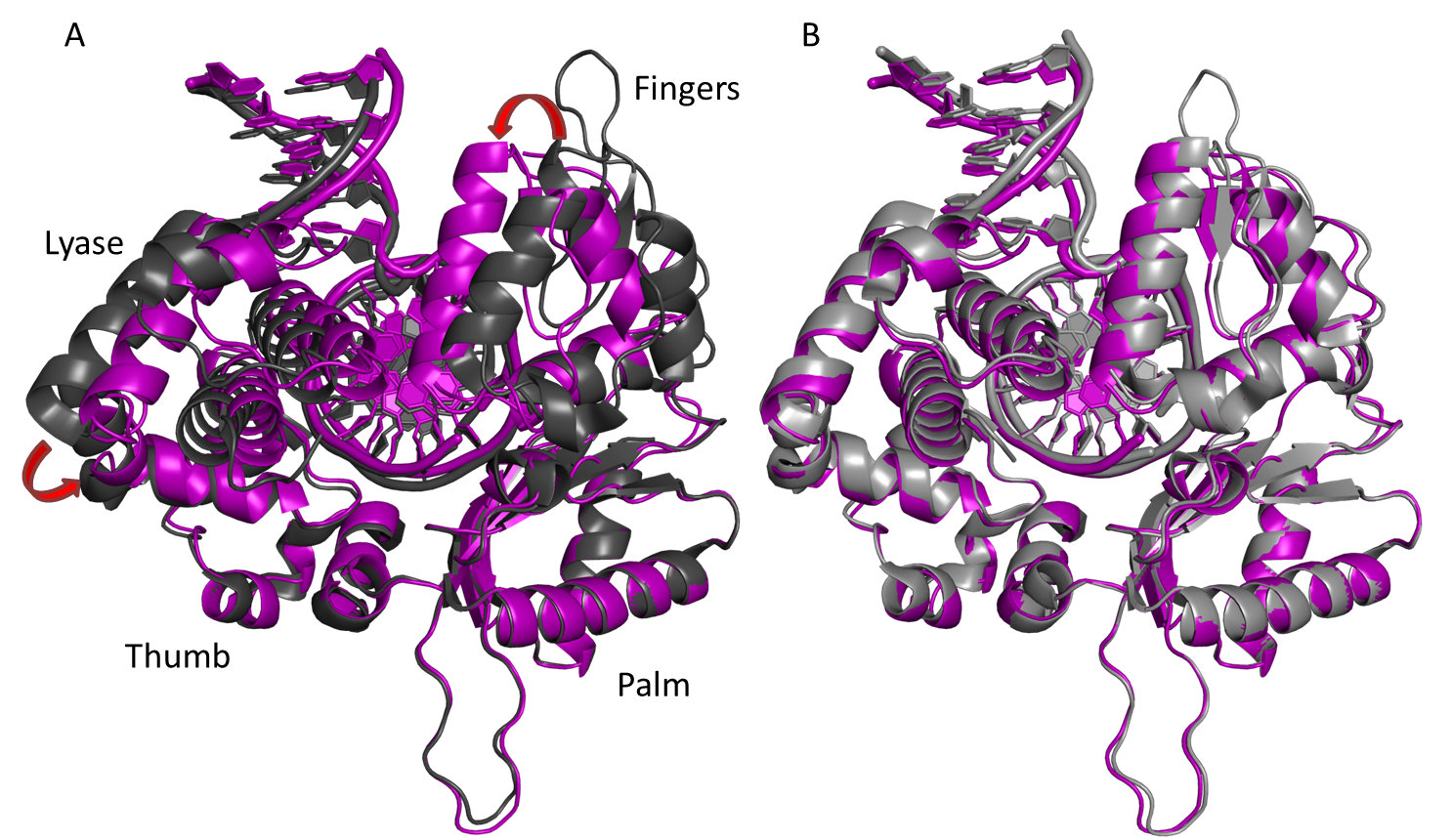
**

**Figure S8A.** Global superpositions of I260Q binary (purple) with WT binary (dark gray, 3ISB) or WT ternary (light gray, 2FMS). **(A)** I260Q and WT binary structures align well in the palm and thumb domains, but not in the lyase and fingers domains, which typically undergo closing movements in the transition from binary to ternary (overall rmsd of 1.22 Å for the polymerase C-α backbone). The DNA substrate also does not align well between the two structures. **(B)** I260Q binary and WT ternary structures align well in all four domains (overall rmsd of 0.49 Å), and the DNA substrate is more closely aligned than in panel A (binary to binary comparison), indicating that the I260Q binary complex favors a prematurely closed state.

**
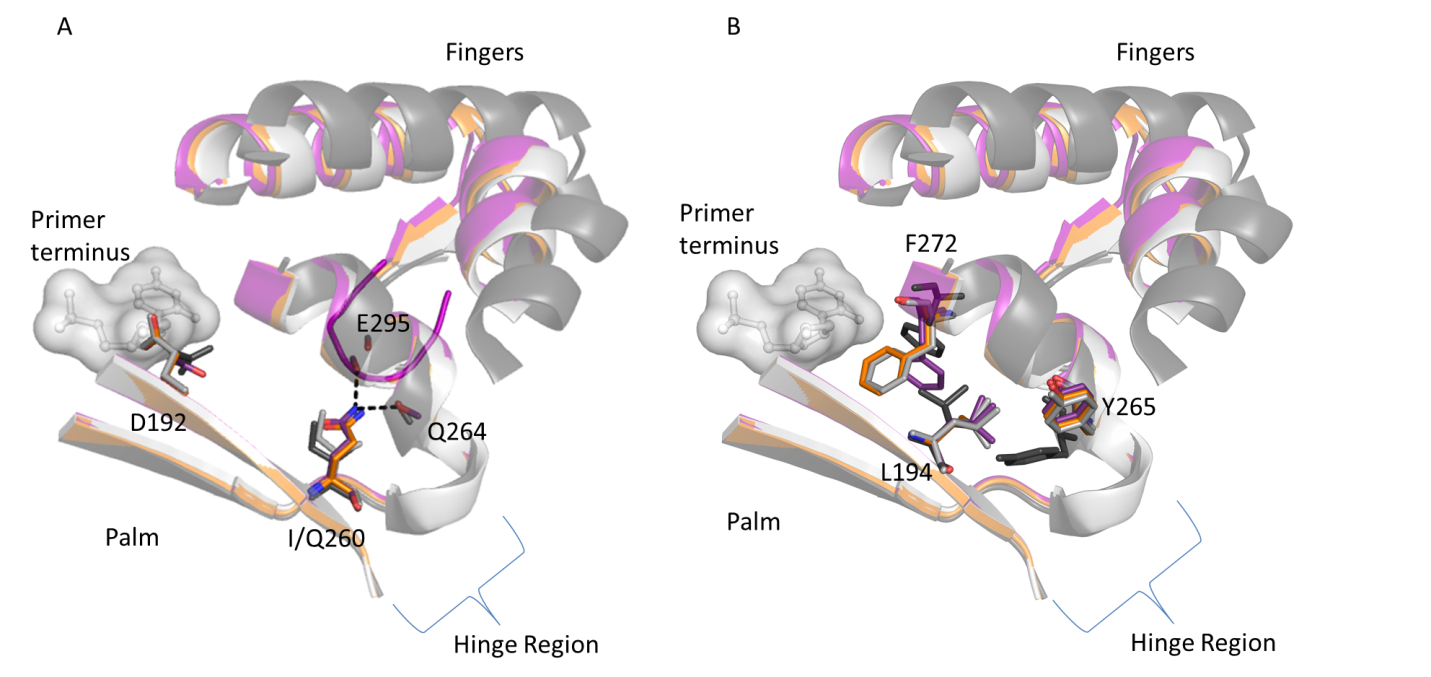
**

**Figure S8B.** Superposition in the hinge region of the I260Q (binary complex purple, ternary complex orange) and WT (binary complex dark gray PDB ID 3ISB*^56^*, ternary complex light gray PDB ID 2FMS*^19^*). (A) Q260 interacts directly with the backbone carbonyl groups of E295 (fingers) and Q264 (hinge-adjacent), pulling the fingers domain and hinge region into ternary-like positions. (B) Hinge residues L194 and Y265 have prematurely moved into ternary positions, while hinge residue F272 remains in an intermediate position.


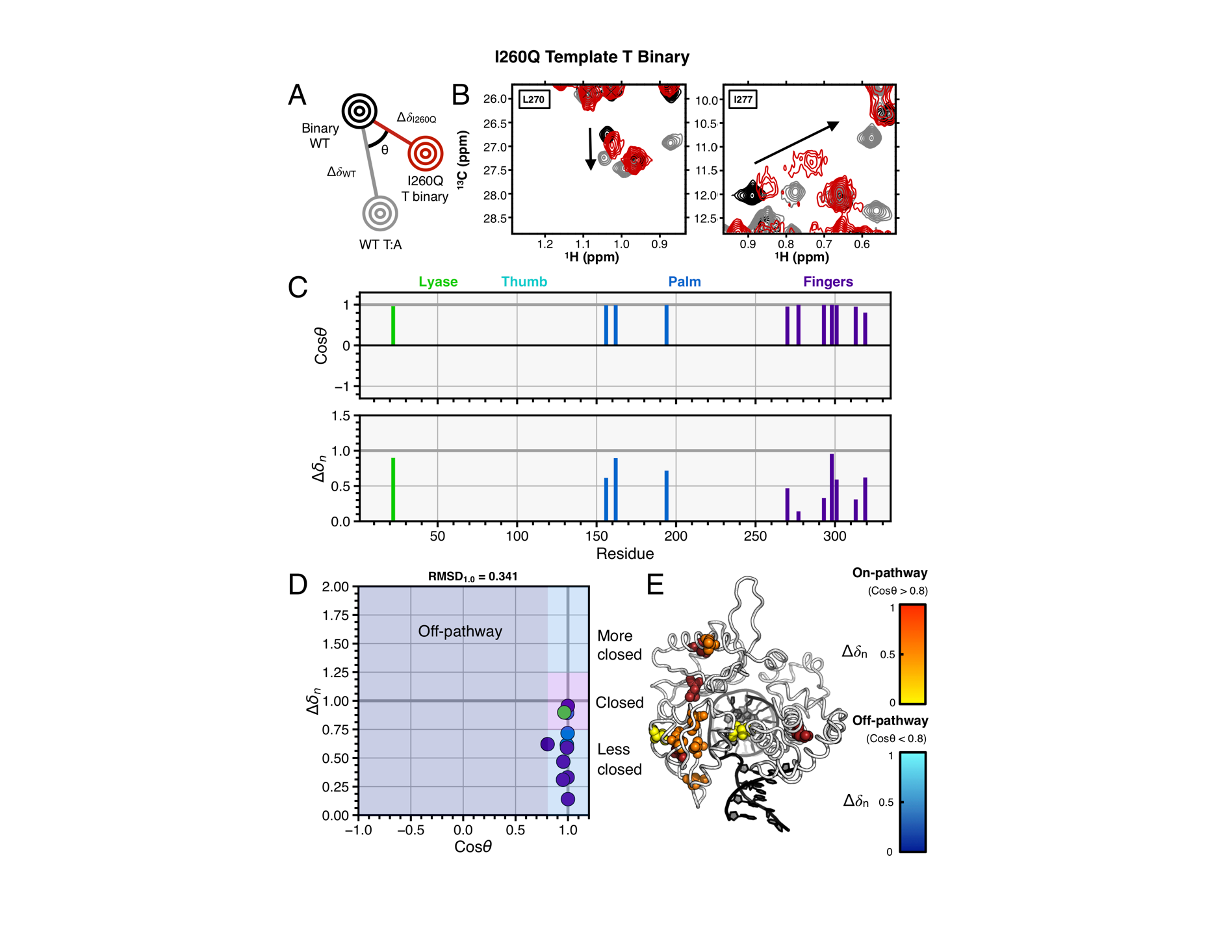


**Figure S9.** **Binary template T I260Q partially closes.** (A) Schematic of the three pol β complexes being compared. δ_I260Q_ refers to the I260Q binary chemical shift magnitude, while δ_WT_ refers to the WT ternary matched chemical shift magnitude. (B) Experimental NMR data for residues L270 and I277 in WT (black and gray) and I260Q (red) pol β. Arrows indicate chemical shift change directionality for enzyme closure. Panel (C) shows the cosθ and chemical shift magnitude (Δδ_n_) from comparison of the WT ternary complex with that of the binary I260Q enzyme as seen in panel (A). The vertical bars are color coded by pol β subdomain, and gray bars indicate cos θ and Δδ_n_ = 1.0. In (D) the cos θ and Δδ_ν_ values are shown, with the combined RMSD for cos θ and Δδ_n_ values from 1.0 shown above the plot. These residues in panel (D) are mapped as spheres on the pol β structure shown in (E). The spheres shown for residues is cos θ > 0.8, considered on-pathway to closure are color coded by chemical shift magnitude with red = 1.0. Blue spheres indicate residues with conformational changes indicative of off-pathway changes.

**
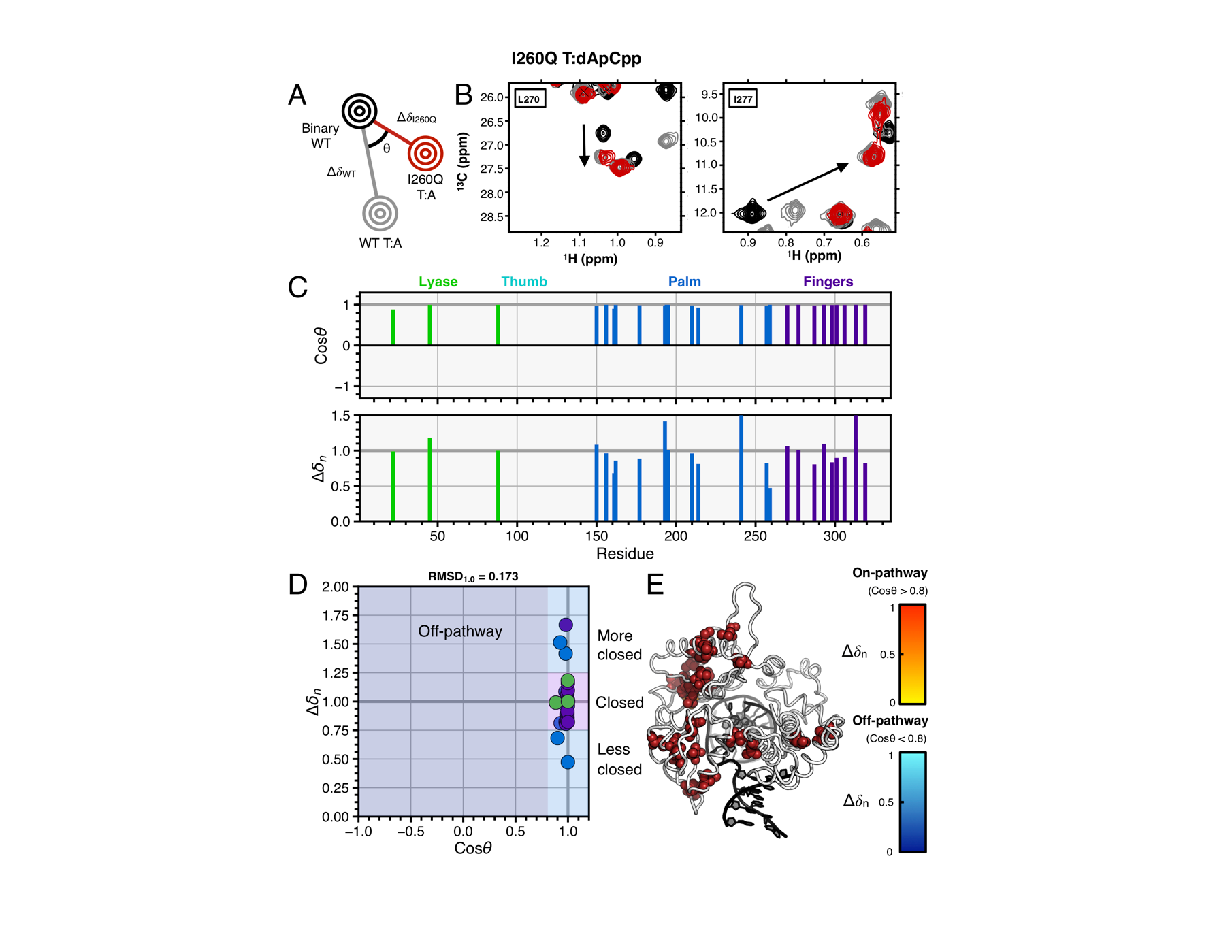
**

**Figure S10. T:dApCpp I260Q.** (A) Schematic of the three pol β complexes being compared. δ_I260Q_ refers to the I260Q T:dApCpp chemical shift magnitude, while δ_WT_ refers to the WT ternary correct chemical shift magnitude. (B) Experimental NMR data for residues L270 and I277 in WT (black and gray) and I260Q (red) pol β. Arrows indicate chemical shift change directionality for enzyme closure. Panel (C) shows the cosθ and chemical shift magnitude (Δδ_n_) from comparison of the WT ternary complex with that of the ternary I260Q enzyme as seen in panel (A). The vertical bars are color coded by pol β subdomain, and gray bars indicate cos θ and Δδ_n_ = 1.0. In (D) the cos θ and Δδ_ν_ values are shown, with the combined RMSD for cos θ and Δδ_n_ values from 1.0 shown above the plot. These residues in panel (D) are mapped as spheres on the pol β structure shown in (E). The spheres shown for residues is cos θ > 0.8, considered on-pathway to closure are color coded by chemical shift magnitude with red = 1.0. Blue spheres indicate residues with conformational changes indicative of off-pathway changes.

**
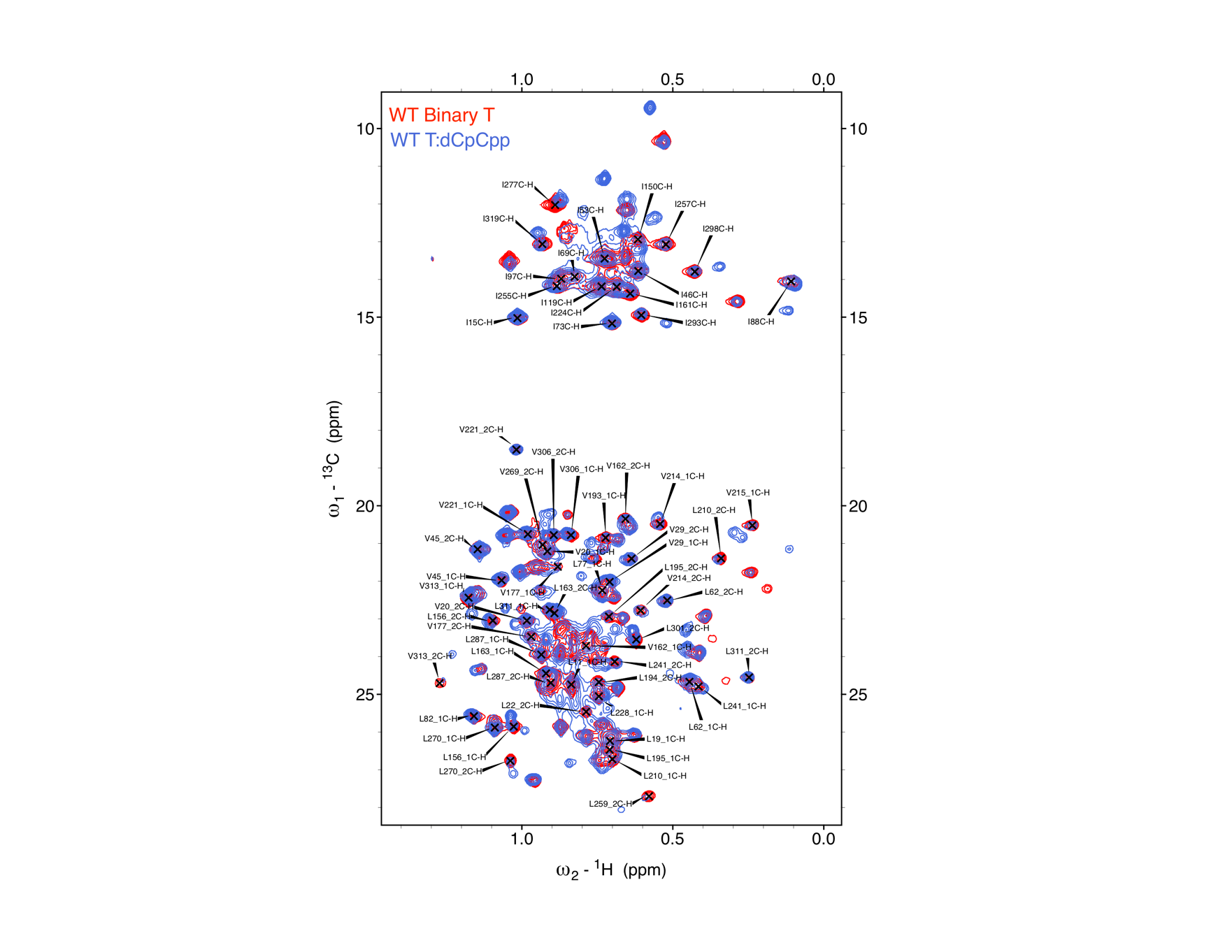
**

**Figure S11. WT T:dCpCpp.** Overlay of WT binary template thymine and T:dCpCpp ILV methyl spectra. Due to the appearance of slow exchange peaks in the T:dCpCpp spectrum, assignment is difficult, and therefore a more qualitative analysis of the T:dCpCpp spectrum is conducted.

**
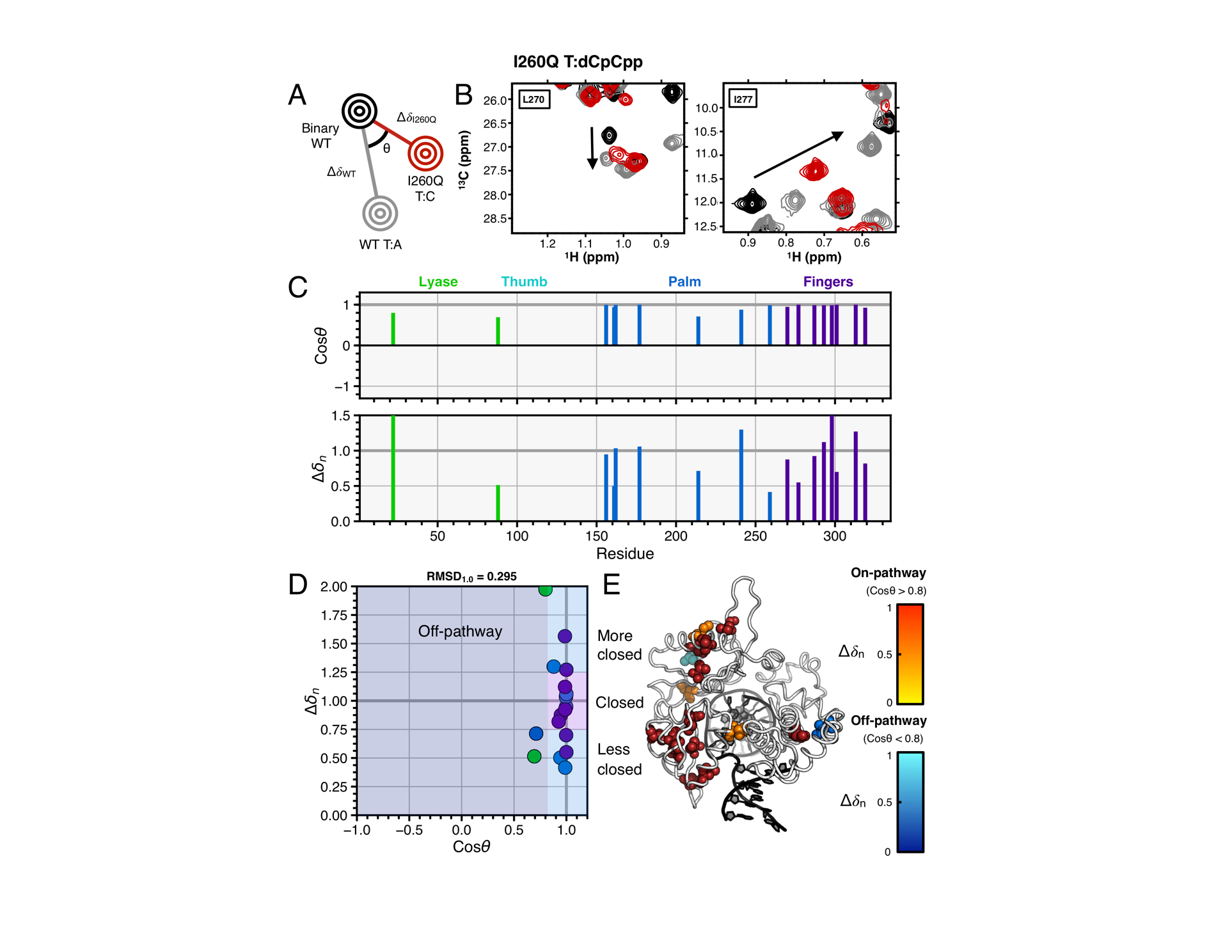
**

**Figure S12. I260Q T:dCpCpp.** (A) Schematic of the three pol β complexes being compared. δ_I260Q_ refers to the I260Q T:dCpCpp chemical shift magnitude, while δ_WT_ refers to the WT ternary correct chemical shift magnitude. (B) Experimental NMR data for residues L270 and I277 in WT (black and gray) and I260Q (red) pol β. Arrows indicate chemical shift change directionality for enzyme closure. Panel (C) shows the cosθ and chemical shift magnitude (Δδ_n_) from comparison of the WT ternary complex with that of the ternary I260Q enzyme as seen in panel (A). The vertical bars are color coded by pol β subdomain, and gray bars indicate cos θ and Δδ_n_ = 1.0. In (D) the cos θ and Δδ_ν_ values are shown, with the combined RMSD for cos θ and Δδ_n_ values from 1.0 shown above the plot. These residues in panel (D) are mapped as spheres on the pol β structure shown in (E). The spheres shown for residues is cos θ > 0.8, considered on-pathway to closure are color coded by chemical shift magnitude with red = 1.0. Blue spheres indicate residues with conformational changes indicative of off-pathway changes.

**
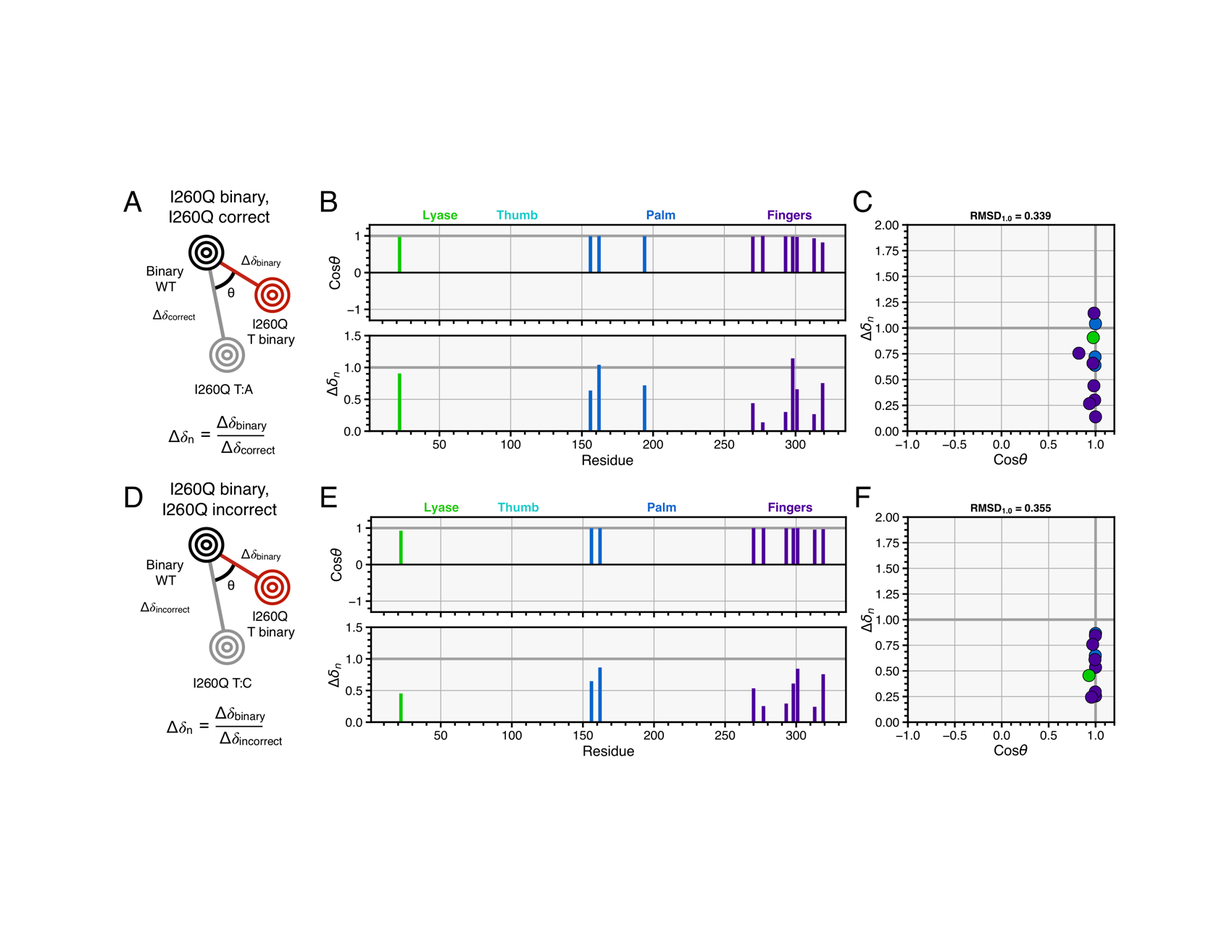
**

**Figure S13. Comparisons between T binary and TA/TC.** Panel A shows vector analysis as described previously, with I260Q binary T compared to the I260Q T:A vector. Panel B shows the cos θ and chemical shift magnitude from comparison of the two ternary complexes with that of the binary enzyme. The vertical bars are color coded by pol β subdomain. In panel C, the cos θ and Δδ_n_ values are shown. Panel D shows vector analysis as described previously, with I260Q binary T compared to the I260Q T:C vector. Panel E shows the cos θ and chemical shift magnitude from comparison of the two ternary complexes with that of the binary enzyme. The vertical bars are color coded by pol β subdomain. In panel F, the cos θ and Δδ_n_ values are shown.


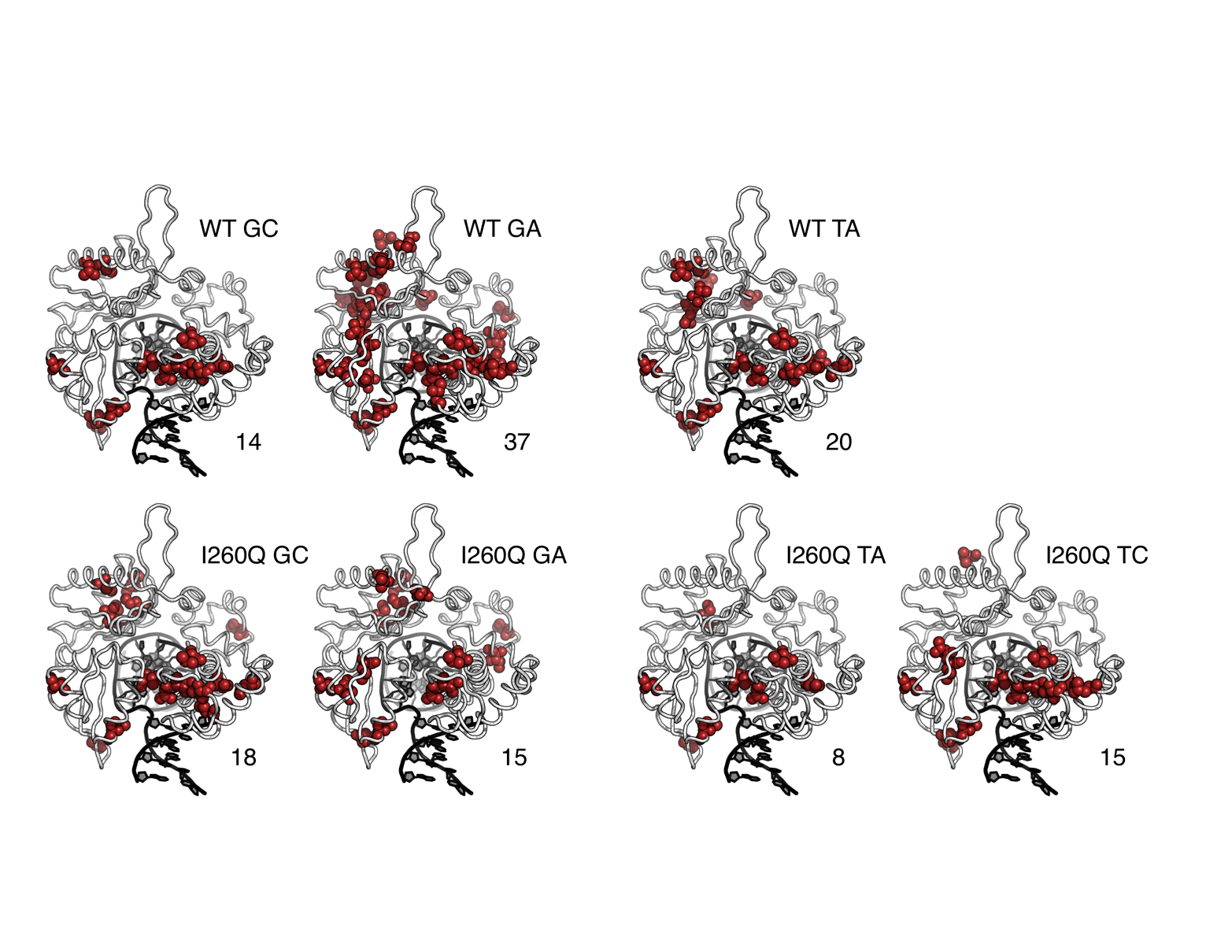


**Figure S14. CPMG Data**. Numbers of flexible residues as observed in CPMG dispersion experiments are given in the bottom right hand of each structure (1BPY). In this representation, cases where two overlapped resonances yield dispersion curves are both considered flexible due to difficulty in distinguishing between assignments.

**Figure S15.** Stopped-flow fluorescence of WT Pol β V303C-AEDANS with incoming correct (A) G:dCTP or (E) T:dATP and I260Q V303C AEDANS with incoming correct nucleotide (B) G:dCTP or (F) T:dATP opposite the templating base G/T, respectively, using extendable DNA. Reverse rates using a trapping experiment for (C) WT ternary with G:dCTP (red) (D) I260Q

ternary with G-dCTP (blue) (G) WT ternary with T:dATP (green); (H) I260Q ternary with T:dATP (magenta). Black lines represent the best fits. See text for descriptions.

**Figure S16.** Stopped-flow fluorescence of WT Pol β V303C-AEDANS and I260Q V303C-AEDANS with ext
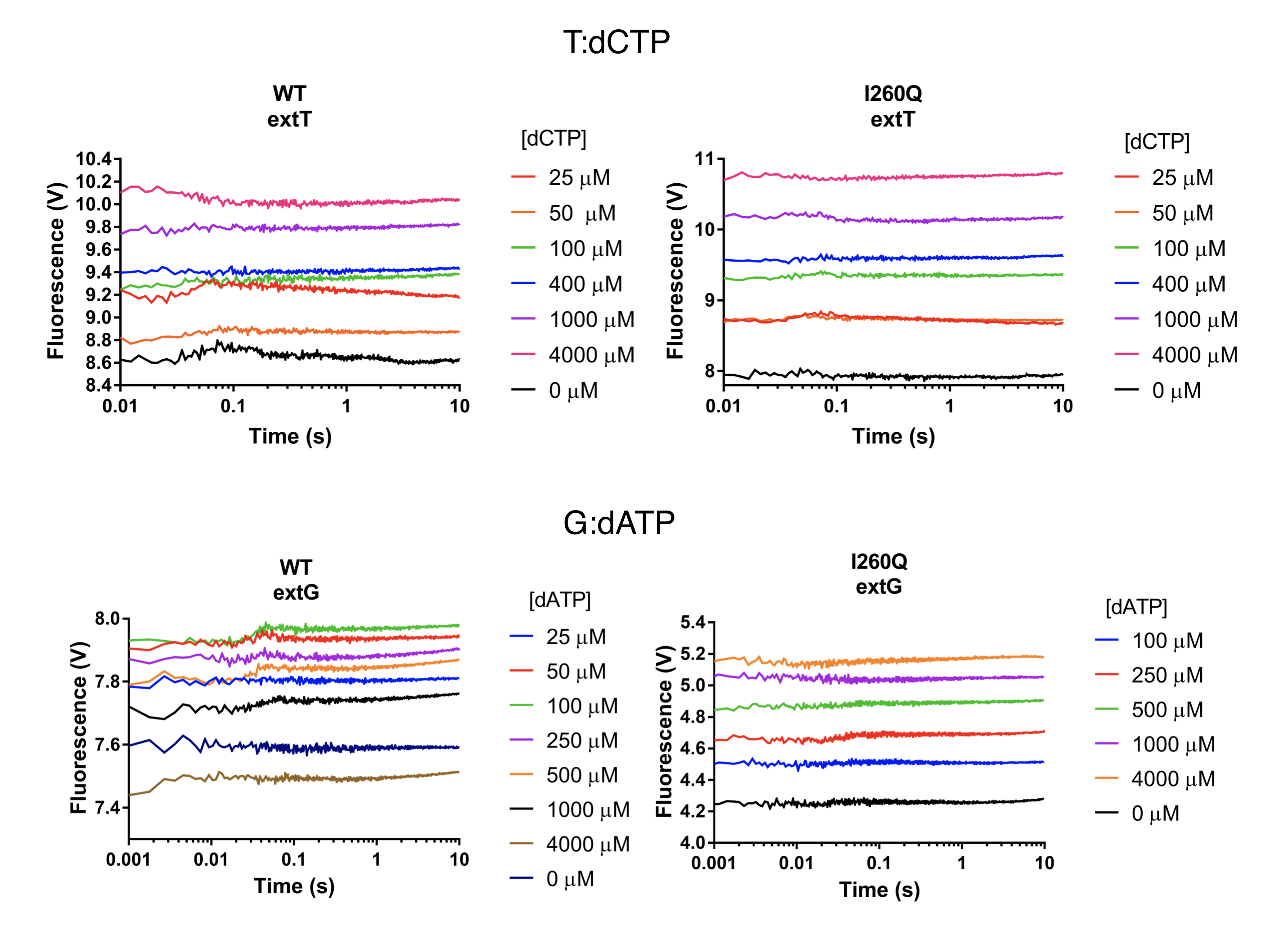
endable DNA with an incoming incorrect dCTP or dATP opposite the templating base T/G respectively.
